# Heterogeneity in endothelial cells and widespread venous arterialization during early vascular development in mammals

**DOI:** 10.1038/s41422-022-00615-z

**Published:** 2022-01-25

**Authors:** Siyuan Hou, Zongcheng Li, Ji Dong, Yun Gao, Zhilin Chang, Xiaochen Ding, Shuaili Li, Yunqiao Li, Yang Zeng, Qian Xin, Baihan Wang, Yanli Ni, Xiaowei Ning, Yuqiong Hu, Xiaoying Fan, Yu Hou, Xianlong Li, Lu Wen, Bin Zhou, Bing Liu, Fuchou Tang, Yu Lan

**Affiliations:** 1 Key Laboratory for Regenerative Medicine of Ministry of Education, Institute of Hematology, School of Medicine, Jinan University, Guangzhou, Guangdong China; 2 State Key Laboratory of Experimental Hematology, Haihe Laboratory of Cell Ecosystem, Institute of Hematology, Fifth Medical Center of Chinese PLA General Hospital, Beijing, China; 3 Integrated Chinese and Western Medicine Postdoctoral Research Station, Jinan University, Guangzhou, Guangdong China; 4 Beijing Advanced Innovation Center for Genomics and Biomedical Pioneering Innovation Center, School of Life Sciences, Peking University, Beijing, China; 5 State Key Laboratory of Proteomics, Academy of Military Medical Sciences, Academy of Military Sciences, Beijing, China; 6 Ministry of Education Key Laboratory of Cell Proliferation and Differentiation, Beijing, China; 7 The State Key Laboratory of Cell Biology, CAS Center for Excellence in Molecular Cell Science, Shanghai Institute of Biochemistry and Cell Biology, Chinese Academy of Sciences, University of Chinese Academy of Sciences, Shanghai, China; 8 Peking-Tsinghua Center for Life Sciences, Peking University, Beijing, China

**Keywords:** Cell biology, Developmental biology

## Abstract

Arteriogenesis rather than unspecialized capillary expansion is critical for restoring effective circulation to compromised tissues in patients. Deciphering the origin and specification of arterial endothelial cells during embryonic development will shed light on the understanding of adult arteriogenesis. However, during early embryonic angiogenesis, the process of endothelial diversification and molecular events underlying arteriovenous fate settling remain largely unresolved in mammals. Here, we constructed the single-cell transcriptomic landscape of vascular endothelial cells (VECs) during the time window for the occurrence of key vasculogenic and angiogenic events in both mouse and human embryos. We uncovered two distinct arterial VEC types, the major artery VECs and arterial plexus VECs, and unexpectedly divergent arteriovenous characteristics among VECs that are located in morphologically undistinguishable vascular plexus intra-embryonically. Using computational prediction and further lineage tracing of venous-featured VECs with a newly developed *Nr2f2*^*CrexER*^ mouse model and a dual recombinase-mediated intersectional genetic approach, we revealed early and widespread arterialization from the capillaries with considerable venous characteristics. Altogether, our findings provide unprecedented and comprehensive details of endothelial heterogeneity and lineage relationships at early angiogenesis stages, and establish a new model regarding the arteriogenesis behaviors of early intra-embryonic vasculatures.

## Introduction

During mammalian embryogenesis, the cardiovascular system is the first functional organ system to form. Vascular endothelial cells (VECs) undergo de novo differentiation from mesodermal precursors and assemble into a primordial vascular network through vasculogenesis. During the establishment of vasculature, VECs are needed to grow new vessels from pre-existing ones via sprouting angiogenesis, while the immature vascular plexus undergoes further remodeling to form recognizable arteries and veins and build a hierarchically organized vascular network.^1–4^ Arteriogenesis refers to the formation of new arteries and arterioles either de novo by means of capillary arterialization or by growth from pre-existing arterial collaterals, which is crucial in embryonic vascular development as well as in adult tissues.^5^ The growth of arteries but not capillaries has been demonstrated to be the key to restoring effective circulation to compromised tissues.^6^ Unfortunately, due to the insufficient understanding of arterial specification and arterial conduit formation, the field of therapeutic angiogenesis has not made significant progress in the past decade. Studies of cellular and molecular events underlying the specification of arterial VECs during embryonic development will help to understand adult arteriogenesis.

In developmental setting, arteries can form in multiple ways,^6^ with cell fate conversion playing a role under certain circumstances. In mice, endocardial cells contribute to a substantial proportion of postnatal coronary arteries through de novo lineage conversion during trabecular compaction.^7^ New arteries can also be generated from venous VECs. During hindbrain vascularization in zebrafish, artery forms by medial sprouting and migration of endothelial cells from a bilateral pair of primitive veins, that is, the primordial hindbrain channels.^8,9^ During mouse development, the coronary arteries of the heart are initially formed by venous VECs from the sinus venosus.^10,11^ In the postnatal mouse retina, vein-derived endothelial tip cells continuously migrate from veins to arterial areas and contribute to emerging arteries.^3,12^ Notably, although the conversion of venous VECs into arteries is observed during the later stages of organogenesis and in tissue regeneration, it is not thought to occur during the expansion of the early vasculature in mammals, when instead branching morphogenesis is generally acknowledged to take place.^3,4,6,10–12^

Recently, single-cell RNA sequencing (scRNA-seq) has been applied to investigate the heterogeneity of endothelial cells in distinct tissues from adult mice.^13–15^ It is proposed that VECs display different properties that correspond to distinct vascular beds^13,14^ and present an arterial-capillary-venous zonation and seamless continuum of transcriptional states.^13,15^ scRNA-seq has also been used to study gastrulation and organogenesis in mouse embryos.^16,17^ Nevertheless, a high-precision and genome-scale gene expression landscape of early embryonic VECs is still lacking. Knowledge of the cellular evolutions and molecular programs underlying the stepwise arteriovenous fate settling will have important implications for developing new approaches for both regenerative and therapeutic purpose. Here, we used scRNA-seq, spanning the time window for the occurrence of key vasculogenic and angiogenic events in both mouse and human embryos,^18,19^ together with genetic lineage tracing, to decipher the endothelial heterogeneity and lineage relationships during early vascular development.

## Results

### Transcriptomic identification of distinct VEC populations in mid-gestational mouse embryos

**Fig. 1:**
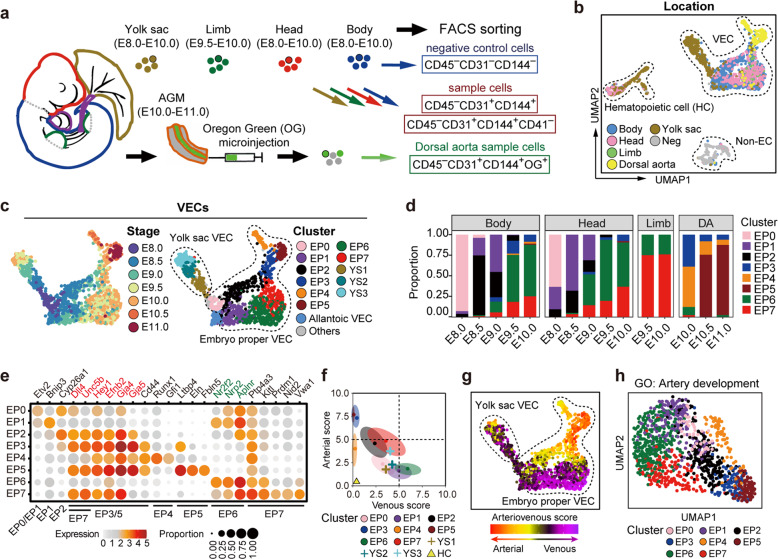
Transcriptomic identification of diverse VEC populations in the mid-gestational mouse embryos. **a** Schematic illustration of strategies used for embryo dissection and cell preparation for the subsequent scRNA-seq. Heart, visceral bud, and umbilical and vitelline vessels outside the embryo proper (purple) were excluded. **b**, **c** UMAP plots showing sampling locations of all sequenced cells (**b**) and stages (**c**) and further yielded twelve clusters (**c**) of all VECs. The main areas of three subtypes of cells (**b**), eight clusters from embryo proper (**c**) and three clusters from yolk sac (**c**) are indicated as closed dashed curves. **d** Bar graph showing the proportion of each VEC cluster at the corresponding locations and stages. DA, dorsal aorta. **e** Dot plot showing the average expression levels and expression proportions of key feature genes distinguishing eight embryo proper VEC clusters. The size of the dot represents the proportion of cells expressing the indicated gene within a cluster, and the color indicates the average expression level of cells within a cluster. Genes in red font indicate the known arterial genes, and those in green font indicate known venous genes. The clusters to which each feature gene belongs are shown at the bottom. Note that embryo proper VEC clusters showed a biased distribution of known arteriovenous markers expect for EP0. **f** Scatterplot showing the average arteriovenous scores of the cells in each cluster. Main distribution ranges of arteriovenous scores in each of the eight embryo proper VEC clusters are also indicated as an oval shape. **g** UMAP plots showing arterial/venous scores of the individual cells in VEC clusters. The main areas of embryo proper VECs and yolk sac VECs are indicated as closed dashed curves. **h** UMAP plot with cells colored by embryo proper VEC clusters. The UMAP coordinates were determined by using 107 genes in the GO term of artery development (GO: 0060840).

We first analyzed mouse embryos from embryonic day (E) 8.0, when most vessel primordia still comprise a series of disconnected clusters of Pecam1-expressing cells,^18,19^ to E11.0, when the first hematopoietic stem cells emerge following endothelial-to-hematopoietic transition.^20,21^ Immunophenotypic endothelial cells (CD45^−^CD31^+^CD144^+^) were collected from different parts of embryos^16,18,22^ (Fig. 1a; Supplementary information, Fig. S1a, b and Table S1). Well-based scRNA-seq^23^ was performed on a total of 2213 cells (Supplementary information, Fig. S1c); 93.4% of these cells passed rigorous quality control checks with no batch effect detected (Supplementary information, Fig. S1d, e). Transcriptomically, VECs were readily distinguished from hematopoietic cells that exhibited considerable expression of *Itga2b* and *Gata1* although they possessed an endothelial immunophenotype^22,24,25^ (Supplementary information, Fig. S2a, b). Hematopoietic cells, together with allantoic VECs that were characterized by the expression of *Hoxa10* and *Hoxa11* and sampled from E8.0–E8.5,^26^ were excluded from the subsequent analyses as they were outside the scope of this work (Supplementary information, Fig. S2a, b and Table S1).

The anatomical distribution of VECs allowed us to assign identities to the two major VEC populations as the embryo proper VEC group and the yolk sac VEC group (Fig. 1b, c; Supplementary information, Fig. S2c–g and Tables S1, S2). Genes that were highly expressed in the embryo proper VECs compared with their expression in the yolk sac VECs were mainly related to neurogenesis and artery development (Supplementary information, Fig. S2h and Table S2), consistent with the notion that VECs and neurons adopt various common mechanisms to control the patterning of vascular and neuronal networks during development.^2^ On the other hand, genes overrepresented in the yolk sac VEC group were predominantly associated with membrane activities, such as cell adhesion and transport (Supplementary information, Fig. S2e–h and Table S2). These findings indicated that embryonic VECs shared features associated with the biological processes of their neighboring cells, suggesting the existence of parenchymal gene signatures during the early development of VECs. Thus, from a transcriptomics point of view, the difference between intra- and extra-embryonic locations was more pronounced than that between different developmental stages when discriminating between embryonic VECs (Fig. 1b, c; Supplementary information, Fig. S2e–h and Tables S1, S2).

The transcriptomically defined embryo proper VECs were further divided into eight clusters (EP0–EP7), the characteristics of which were inferred by their spatiotemporal distribution and the expression of key genes^10,27–36^ (Fig. 1b–e; Supplementary information, Fig. S2i–k and Tables S1, S3). EP0 mainly contained cells from E8.0 embryos, indicative of its features of primordial VECs (Fig. 1b–e). Although these earliest VECs may originate independently, their molecular characteristics were similar. Using the known arterial and venous genes, we scored the arteriovenous features of all the VEC populations (Fig. 1f, g). Hematopoietic cells were used as a negative control as they were expected to lack both arterial and venous features^36^ (Fig. 1f). None of the populations showed both apparent arterial and venous features (Fig. 1f), in line with the notion that arterial and venous fates are mutually exclusive.^37^ EP1 and EP6 manifested different degrees of venous characteristics, whereas four clusters (EP2, EP3, EP5, and EP7) displayed arterial characteristics to different degrees (Fig. 1f, g). Importantly, the clusters of embryo proper VECs could be readily distinguished in the specifically reconstituted Uniform Manifold Approximation and Projection (UMAP) by only using the genes included in the Gene Ontogeny (GO) term ‘artery development’ (Fig. 1h). We also evaluated the arteriovenous manifestations of yolk sac VECs, and found that arterial- or venous-featured cells were much fewer than in embryo proper VECs at all the comparable developmental stages (Supplementary information, Fig. S2l). The further yielded three clusters of yolk sac VECs were mainly related to their developmental stages rather than arteriovenous features (Fig. 1c, f, g; Supplementary information, Fig. S2m, n). Therefore, arteriovenous characteristics made a considerable contribution to the identities of distinct VEC populations in the embryo proper but not yolk sac. We also validated the higher expression of endothelial CD36 and Neurl3^36^ in the yolk sac of late stage (E9.5–E10.0) than in early stage (E8.5), in accordance with the transcriptomic finding (Supplementary information, Fig. S2o, p).

To determine whether the VEC populations we had identified could be readily recognized using other sequencing strategies, we performed droplet-based (10× Genomics) scRNA-seq. A total of 10,465 immunophenotypic endothelial cells from 44 embryos at E9.0–E10.0 passed rigorous quality control measures and no batch effect was detected (Supplementary information, Fig. S3a–c and Table S4). Unsupervised clustering yielded eight clusters (Supplementary information, Fig. S3d and Table S4). Of these, five clusters, which comprised the majority of the sampled cells (96.9%), exhibited early or arteriovenous VEC characteristics (Supplementary information, Fig. S3d, e). The other three clusters were less relevant and were thus excluded from the subsequent analyses. One of these clusters was a *Gata4*-expressing population, which likely involved cardiac (*Hand2*^+^) and liver (*Oit3*^+^) VECs,^13,17,38^ presumably due to accidental incorporation during the dissection of embryos, as the heart and visceral bud should be prospectively excluded before sampling (Supplementary information, Fig. S3d–g). The features of the five major clusters corresponded closely with the clusters identified by the well-based scRNA-seq, among which Vwa1^+^ VEC population was transcriptomically similar to EP7, with both expressing a set of arterial genes along with several specific genes, such as *Vwa1* and *Nid2* (Fig. 1e; Supplementary information, Fig. S3e, h). Principal Component Analysis (PCA) recapitulated the result obtained via UMAP, with the top genes of the PC1 axis including several known arterial markers, validating the arterial properties of the arterial VEC and Vwa1^+^ VEC clusters (Supplementary information, Fig. S3h, i). Taken together, our findings showed that identification of the principal VEC populations was reproducible using different sequencing strategies. However, given the relatively limited resolution and lack of spatiotemporal information resulting from the droplet-based scRNA-seq, we returned to the well-based scRNA-seq data for the subsequent analyses.

### Spatiotemporal localization of distinct embryonic VEC populations

**Fig. 2:**
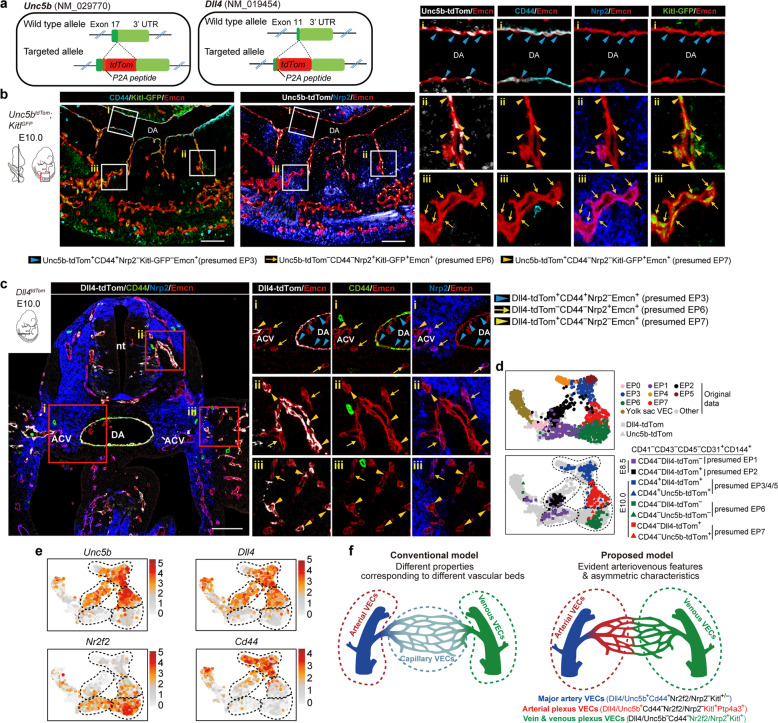
Anatomical localization of distinct VEC populations in the embryo proper. **a** Schematic models of the gene-targeting strategies for generating *Unc5b-tdTomato* (left) and *Dll4-tdTomato* (right) reporter mouse lines via CRISPR/Cas9 system. Note that the *tdTomato* cDNA was introduced between the last exon and the 3' UTR of mouse *Unc5b* or *Dll4* genes to ensure that tdTomato was expressed in exactly the same way as the labeled genes without abolishing their function. **b** Representative immunostaining on sagittal sections of E10.0 *Unc5b-tdTomato;Kitl-GFP* embryos at body part. Note that the localization of major artery VECs (Unc5b-tdTom^+^CD44^+^Nrp2^−^Kitl-GFP^−^Endomucin^+^, blue arrowheads) at known arteries such as the dorsal aorta, EP7 VECs (Unc5b-tdTom^+^CD44^−^Nrp2^−^Kitl-GFP^+^Endomucin^+^, yellow arrowheads) at intersegmental arteries and vascular plexus, and EP6 VECs (Unc5b-tdTom^−^CD44^−^Nrp2^+^Kitl-GFP^+^Endomucin^+^, yellow arrows) at vascular plexus adjacent to EP7. Unc5b^+^ arterial VECs and Nrp2^+^ venous VECs displayed a largely complementary expression pattern, and they comprised most VECs in mid-gestational embryos. Images in white boxes are shown at high magnification. The diagrams on the left indicate the positions of sections and imaging. DA, dorsal aorta. Scale bars, 100 μm. **c** Representative immunostaining on cross sections of E10.0 *Dll4-tdTomato* embryos at body part. Note the localization of major artery VECs (Dll4-tdTom^+^CD44^+^Nrp2^−^Endomucin^+^, blue arrowheads) at known arteries such as DA, EP6 VECs (Dll4-tdTom^−^CD44^−^Nrp2^+^Endomucin^+^, yellow arrows) at vascular plexus and known veins such as ACVs, and EP7 VECs (Dll4-tdTom^+^CD44^−^Nrp2^−^Endomucin^+^, yellow arrowheads) at vascular plexus adjacent to EP6. Images in red boxes are shown at high magnification. The diagram on the upper left indicates the position of sections. nt, neural tube; ACV, anterior cardinal vein; DA, dorsal aorta. Scale bars, 100 μm. **d** UMAP plots combining VECs from our original data and those from arterial reporter mouse models including *Dll4-tdTomato* and *Unc5b-tdTomato* embryos. Note that the indicated marker combinations at given developmental stages effectively distinguish the in silico-identified main VEC populations, which are outlined in dashed curves. **e** UMAP plots showing the expression levels of indicated arteriovenous marker genes. Dashed outlines of different VEC populations are also indicated. **f** Schematic diagram of the conventional model (left) and proposed model in the present study (right) illustrating the molecular characteristics of early intra-embryonic vasculatures. The dashed circles in red and green correspond to arterial and venous VECs, respectively.

To delineate the exact localization of each VEC cluster in the embryo proper, we specifically generated two pan-arterial VEC reporter mouse lines, *Unc5b*^*tdTomato*^ and *Dll4*^*tdTomato*^, using a CRISPR/Cas9-mediated gene knockin strategy, and both expression was further validated on the postnatal retina^39–41^ (Fig. 2a; Supplementary information, Fig. S4a). We then developed a set of marker combinations to delineate the exact localization of principal VEC populations identified here (Supplementary information, Fig. S4b). EP1 and EP2, the two main clusters identified at E8.5 lacked Kitl expression. Anatomically, EP2 was located exclusively at major arteries, including the dorsal aortae and internal carotid arteries at E8.5, and was thus recognized as early arterial VECs (Fig. 1d, e; Supplementary information, Fig. S4b, c). In comparison, EP1 showed a vascular plexus distribution and was designated as early plexus VECs (Supplementary information, Fig. S4b, c). EP3 and EP5 were localized at dorsal aortas and their first-order side branches at E9.5–E10.5 (Fig. 2b, c; Supplementary information, Fig. S4d–f), with the relatively high Ltbp4^+^ signals at later stage indicative of EP5 characteristics (Supplementary information, Fig. S5a). Thus, the development from EP2 to EP3 and EP5 represented the gradual cellular progression of major artery structures.

EP6 mainly came from E9.5–E10.0 and was characterized by the expression of the venous VEC marker Nrp2 in addition to Kitl, but not arterial VEC markers (Fig. 1d, e; Supplementary information, Fig. S4b). EP6 was localized at known venous structures, such as the anterior cardinal veins, and also abundantly present in the nearby capillary plexus (Fig. 2b, c; Supplementary information, Fig. S4d–f). It was thus identified as vein & venous plexus VECs. Distributed at the same developmental stages as EP6, EP7 expressed several known arterial genes in addition to *Kitl*, such as *Dll4*, *Unc5b*, *Hey1*, *Efnb2*, and *Gja4*, but a low level of major artery genes, such as *Gja5* and *CD44*, and venous VEC genes, such as *Nrp2* and *Nr2f2* (Fig. 1d, e; Supplementary information, Fig. S4b). EP7 served as the anatomical extension of the EP3 arterial structure, generally being located at the distal branches of major arteries, and was adjacent to the EP6 plexus in the form of small vessels widely distributed throughout the whole embryo, including the perineural vascular plexus, vascular sprouts within the neural tube, intersegmental vessels at the body trunk, and capillaries in the limb buds (Fig. 2b, c; Supplementary information, Figs. S4d–f and S5b). Therefore, EP7 was identified as arterial plexus VECs.

Furthermore, we isolated different endothelial populations from both *Dll4*^*tdTomato*^ and *Unc5b*^*tdTomato*^ reporter mouse lines using the same marker combinations as those used for immunostaining, and performed scRNA-seq (Supplementary information, Fig. S5c and Table S5). Importantly, when projected onto the original UMAP, the newly sequenced VEC populations clustered together with the cell populations that they were presumed to be (Fig. 2d, e). We also validated previously unknown markers of two arterial-featured clusters with the use of RNAscope. As a marker gene of early arterial VECs (EP2) (Fig. 1e; Supplementary information, Fig. S2k), endothelial Cyp26a1 expression was confined to the aortae at E8.5 and was absent at E10.0 (Supplementary information, Fig. S5d). *Ptp4a3* was transcriptionally activated in arterial plexus VECs (EP7) (Fig. 1e), which, anatomically, showed a predominantly endothelial expression pattern. The location was restricted to some small vessels, including intersegmental branches from the dorsal aorta, in accordance with that of EP7, identified by the known arterial markers Dll4 or Unc5b (Supplementary information, Fig. S5d).

Taken together, two types of arterial VECs, namely the major artery VECs (EP3 and EP5) and arterial plexus VECs (EP7) could be readily discriminated, whereas the morphologically well-defined major veins and venous plexus were largely indistinguishable on a molecular basis. The asymmetric arteriovenous characteristics are different to those seen in adults, where VECs display different properties that correspond to distinct vascular beds^13,15^ (Fig. 2f).

### Molecular characteristics and mural cell properties of different arterial vasculatures in the embryo proper

**Fig. 3:**
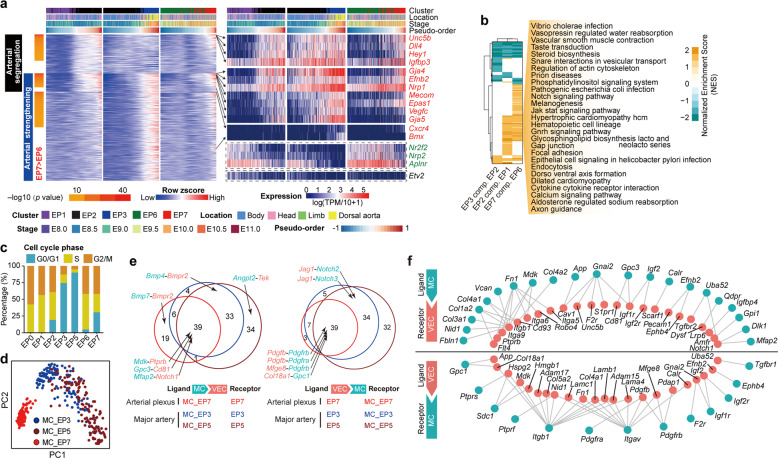
Molecular evolution and predicted VEC–mural cell interactions of different arterial vasculatures in the embryo proper. **a** Heatmaps showing the dynamic relative expression levels of pattern genes in EP1, EP2 and EP3 with the corresponding relative expression levels in EP6 and EP7 (smoothed over 20 adjacent cells). Cells are ordered by pseudo-order axes inferred in Supplementary information, Fig. S6a. Genes are ordered firstly by pattern categories and then by *P* values of comparison between EP6 and EP7. Expression levels of selected arterial genes from the pattern genes (red font), known venous genes (green font), and the primordial VEC maker *Etv2* are shown on the right. **b** Heatmap showing the normalized enrichment scores (NES) of KEGG pathways by GSEA analysis between each two spatially or temporally adjacent embryo proper VEC clusters. Only significantly changed pathways (FDR < 0.1) in at least one pair of comparison are shown. Row and column dendrogram are generated by using hierarchical clustering of significance indicators (1, significantly up-regulated; 0, not significantly changed; −1, significantly down-regulated between the indicated pairs) with Ward’s linkage algorithm and Euclidean distance measure. Shared up-regulated pathways by two types of arterial VEC segregation (EP2 compared with EP1 and EP7 compared with EP6) are noted on the right. **c** Bar charts showing the constitutions of cells with different cell cycle phases in the indicated embryo proper VEC clusters based on molecular signatures in the scRNA-seq data. **d** PCA of mural cell populations. Mural cells with different derivations are shown in different colors. Note that MC_EP7 was separated from the other two mural cell populations in the dimension reduction maps, indicating their distinct molecular features. MC, mural cells. **e** Venn diagrams showing the numbers of shared and distinct heterologous ligand–receptor pairs between different arterial VEC populations and their corresponding mural cell populations. Representative ligand–receptor pairs are indicated. MC, mural cells. **f** Networks of shared heterologous ligand–receptor pairs among three arterial vasculatures. The panel above shows gene network with MC as ligand and VEC as receptor, and the panel below shows gene network with VEC as ligand and MC as receptor. MC, mural cells.

We next investigated changes in gene expression across spatially or temporally continuous VEC populations. The pseudo-order within EP1 and EP2 inferred a continuum of arterial–venous identity on the initially established circulatory loop, whereas that within EP2 and EP3 represented the molecular changes occurring in major arteries during their development (Fig. 3a; Supplementary information, Fig. S6a and Table S3). We revealed three main expression patterns which respectively represented changes in segregation into the first arterial fate and/or in the subsequent strengthening of arterial features (Fig. 3a; Supplementary information, Table S3), and found that the expression of most arterial segregation-related genes was also elevated in EP7 compared with their expression in EP6, with many acknowledged arterial markers highly ranked (Fig. 3a; Supplementary information, Table S3). The similarity between these two types of arterial fate segregation, namely the initial major artery fate settling (EP2 as compared with EP1) and arterial fate segregation in the vascular plexus (EP7 as compared with EP6) was further supported by their considerable sharing of significantly enriched signaling pathways (Fig. 3b; Supplementary information, Table S3), top differentially expressed genes and transcription factors, and enriched terms (Supplementary information, Fig. S6b–g). Cell cycle analysis of distinct VEC populations demonstrated an increase in quiescent status accompanied by arterial feature segregation, strengthening, and maturation, including when EP7 was compared with EP6 (Fig. 3c). This was in accordance with previous findings showing the enabling role played by cell cycle suppression in arterial differentiation.^10,42,43^ The different proliferation status of distinct VEC populations was also validated by flow cytometric evaluation of Ki67 expression and BrdU incorporation with subsequent immunostaining (Supplementary information, Fig. S6h–j).

Different arterial VEC populations showed distinct mural cell coverage status around E10. PDGFRβ-expressing mural cells were distributed among all the arterial vasculatures that we identified (major arteries and the arterial plexus), whereas α-SMA-expressing mural cells were restricted to the major arteries (Supplementary information, Fig. S7a, b). We further isolated PDGFRβ-expressing mural cells around the dorsal aorta at E9.5 and E10.5 and in limb buds at E9.5,^44^ corresponding to three different arterial populations including two major artery VEC populations EP3 and EP5 and arterial plexus VECs EP7, respectively, and performed scRNA-seq (Fig. 3d; Supplementary information, Fig. S7a–c and Table S6). Of note, most of the heterologous ligand–receptor pairs in the arterial plexus were shared with those in the major arteries, implying largely similar mechanisms underlying the arterial stabilization mediated by mural cell–VEC interactions, irrespective of the vascular beds (Fig. 3e, f; Supplementary information, Fig. S7d, e and Table S6). More potential interactions were found in major arteries than in the arterial plexus, with E10.5 having the highest number, suggesting enhanced mural cell–VEC interactions during artery maturation (Fig. 3e, f; Supplementary information, Fig. S7e and Table S6).

### Transcriptomic identification of two types of arterial VEC populations in human embryos

To determine whether our finding with respect to the two types of embryonic arterial VECs is conserved among mammals, scRNA-seq was performed with immunophenotypic endothelial cells derived from different intra-embryonic sites of totally five human embryos that were spatiotemporally comparable to mouse embryos used in this study^45,46^ (Supplementary information, Fig. S8a, b and Table S7). A total of 835 of 966 (86.4%) sampled cells passed a strict quality control assessment, and the batch effect and cell cycle effect were regressed out (Supplementary information, Fig. S8b, c and Table S7). Similar to the result of 10× Genomics scRNA-seq of mouse VECs (Supplementary information, Fig. S3d–g), a *GATA4*^*+*^*HAND2*^*+*^ and a *GATA4*^*+*^*OIT3*^*+*^ cluster, possibly corresponding to cardiac and liver endothelial cells, respectively, were also identified^38,47^ (Supplementary information, Fig. S8d, e). These two minor clusters, together with a hematopoietic cell cluster featured by the expression of erythroid cell marker *GYPA* and the presumed hemogenic endothelial cells characterized by *RUNX1* expression, were excluded; the two remaining vascular VEC clusters of interest were retained for further analyses.^46^

**Fig. 4:**
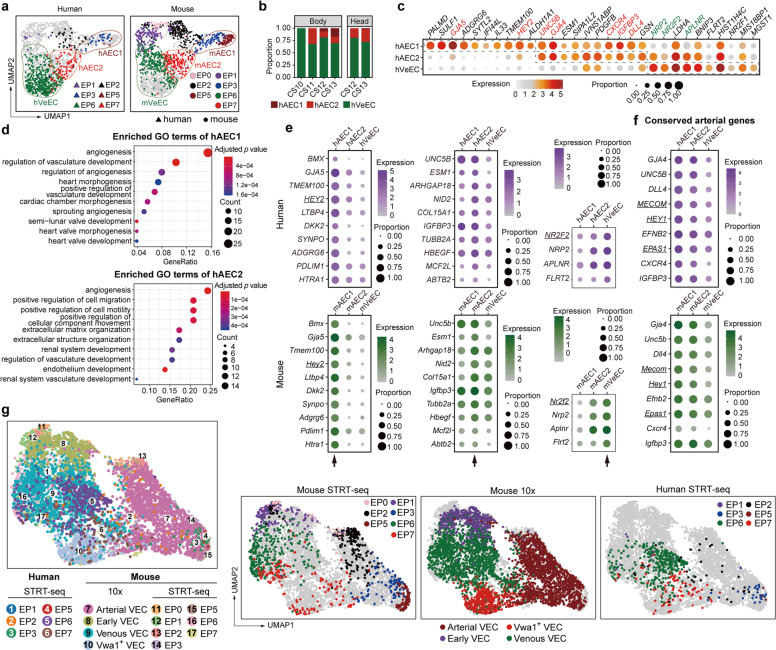
Transcriptomic identification of different arterial VEC populations in human embryos. **a** UMAP plots showing integrated analysis of VECs from mouse and human embryos by STRT-seq. The human EP3 and EP5 combination was renamed as human arterial VEC1 (hAEC1), EP7 as hAEC2, and EP6 as human venous VEC (hVeEC), corresponding to the mouse EP3 and EP5 combination (mAEC1), EP7 (mAEC2), and EP6 (mVeEC), respectively. **b** Bar graph showing the proportion of each human cluster at the corresponding locations and stages. CS, Carnegie stage. **c** Dot plot showing the expression levels of top ten marker genes for each of the three indicated human VEC populations. Note that hAEC1 and hAEC2 shared several classical arterial genes, whereas hAEC1 specifically overrepresented *GJA5* and *HEY2*. Known arterial and venous genes are indicated in red and green, respectively. **d** The top ten enriched GO biological process terms of hAEC1 (upper) and hAEC2 (lower). Dot color indicates the statistical significance of the enrichment and dot size represents the number of genes annotated to each term. **e**, **f** Dot plots showing the average expression level and expression percentage of the top ten (all included if less than ten) conserved genes for each of the three types of arteriovenous VECs as indicated by arrows (**e**) and those of the nine conserved arterial genes, when defined as significantly overrepresented in all the four comparisons (two arterial clusters respectively compared to the corresponding venous cluster in both human and mouse) (**f**). Size of the dot represents the percentage of cells expressing the indicated gene within a cluster, and color indicates the average expression level of the gene within a cluster. Genes are listed in ascending order of Fisher’s combined *P* value from independent *P* values of human and mouse datasets. Genes encoding transcription factors are underlined. **g** UMAP plots showing integrated analysis of three embryonic VEC-related datasets from human and mouse embryos and sequenced by using STRT-seq and 10× Genomics methods.

Human embryonic VECs were combined with mouse embryo proper VECs identified by the same sequencing strategy for the integration and comparison analysis (Figs. 1b, c, 4a; Supplementary information, Fig. S8d, e and Table S7). The assignment accuracy was further confirmed by correlation analysis in Mutual Nearest Neighbors (MNN) Space (Supplementary information, Fig. S8f). Some human VECs were clearly assigned to the two types of arterial-featured VECs, namely major arterial VECs EP3 and EP5, which were together renamed as AEC1 to simplify the annotation, and arterial plexus VECs EP7, which were renamed hereafter as AEC2 (Fig. 4a–c; Supplementary information, Fig. S8f, g and Table S7). Both human AEC populations showed a higher proportion of cells in a quiescent status compared with the venous-featured VEC population EP6 (renamed hereafter as VeEC), with AEC1 being the most quiescent (Supplementary information, Fig. S8h). In addition, enriched terms of the highly expressed genes in human AEC2 but not AEC1 showed characteristics that suggested a propensity toward cell migration and motility (Fig. 4d).

We next determined conserved arteriovenous feature genes in these two species during vascular development. A total of 68 and 10 conserved genes were screened out for major arterial VECs (AEC1) and arterial plexus VECs (AEC2), respectively (Fig. 4e; Supplementary information, Fig. S8i and Table S7). On the other hand, only four genes were screened out as conserved genes for the embryonic venous-featured VEC population, including the known venous markers *NR2F2*, *NRP2*, and *APLNR* (Fig. 4e). We also made an effort to identify conserved arterial genes, which were defined as those highly expressed in both AEC populations when respectively compared with the corresponding venous VEC population. A total of 20 conserved arterial genes were identified, with the 9 most conserved ones being well-known arterial genes, including three transcription factors, *MECOM*, *HEY1*, and *EPAS1*, as well as *UNC5B* and *DLL4* (Fig. 4f; Supplementary information, Fig. S8j), the homologs of which in mouse were used as pan-arterial markers for the in situ validation in this study (Figs. 1e and 2b, c; Supplementary information, Figs. S4c–f and S5b).

Finally, an integrated single-cell intra-embryonic VEC map within the time window of our interest was constructed, which included the datasets of both mouse and human embryonic VECs developed here, using both well-based and droplet-based sequencing strategies. This will act as a resource to facilitate future studies of early vascular development in mammals (Fig. 4g; Supplementary information, Fig. S8k and Table S7).

### Evolutionarily conserved developmental paths of two types of arterial VECs predicted by trajectory analyses

**Fig. 5:**
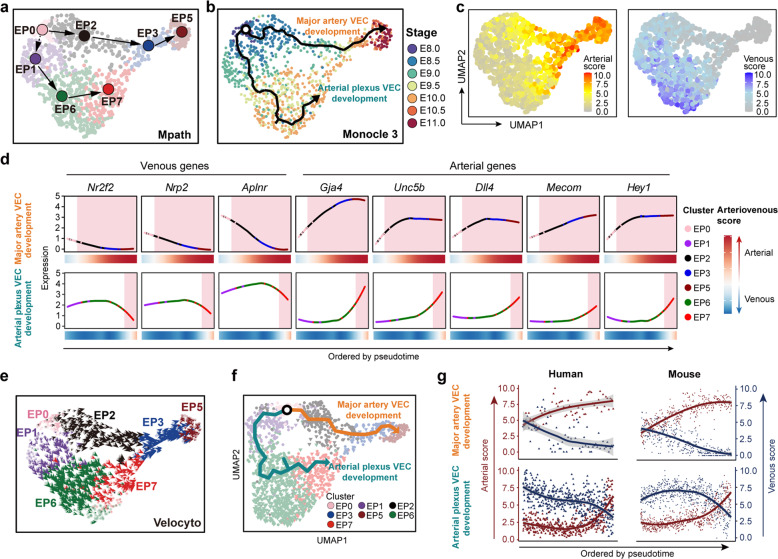
Computational analysis predicted the basically conserved development paths of two types of arterial VECs between human and mouse. **a** Developmental trajectory among embryo proper VEC clusters inferred by Mpath. The directional arrows are inferred by Mpath and sampling stages. **b** Trajectory of cells in embryo proper VEC clusters inferred by monocle 3 showed two major directions mapped with sampling stages, which largely recapitulated the actual temporal order of the sampled cells. **c** UMAP plots showing arterial (left) and venous (right) scores of the individual cells in the embryo proper VEC group. **d** Loess regression-smoothened expression of the indicated venous and arterial marker genes along two distinct arterial VEC development paths inferred by Monocle 3. Smoothened arteriovenous scores are also shown at the bottom. Red shading, arterial-featured VEC populations. **e** Velocity vector field displayed on the UMAP plot at single-cell level, with each arrow colored by cluster showing the movement direction and speed of each individual cell, indicating two waves of arterial differentiation, one of which was presumably derived from vein & venous plexus VECs (EP6). **f** UMAP plot showing the trajectory inferred by Monocle 3, which identified two predominant paths from primordial VECs. **g** Scatter plots showing arterial (red) and venous (blue) scores of the cells along major artery VEC development (upper) and artery plexus VEC development (lower) paths, with loess-smoothed fit curves indicated. VECs from human and mouse embryos are illustrated separately, and cells are ordered by pseudotime.

Mpath^48^ predicted two developmental paths of VECs from primordial VECs (EP0). One was toward mature arterial VECs (EP5), experiencing early arterial VECs (EP2) and the subsequent maturing arterial VECs (EP3), which represented the step-by-step cellular changes of major artery VECs. The other was toward early plexus VECs (EP1) and further vein & venous plexus VECs (EP6), with the final fate as arterial plexus VECs (EP7), reflecting the cellular evolution of the vascular plexus (Fig. 5a). Trajectory analysis by Monocle 3 at single-cell resolution^17^ largely recapitulated the actual temporal order of the sampled cells and showed two major directions (Fig. 5b). In general, two waves of arterial fate were sequentially segregated out. One came directly from primordial VECs, known as via vasculogenesis, and the other involved the branching of arterial plexus VECs from the venous-featured VEC population, through angiogenesis and remodeling (Fig. 5b–d; Supplementary information, Table S1). We also performed RNA velocity analysis to predict the future state of each individual cell, based on an algorithm that was distinct from other trajectory analyses.^49^ The results were consistent with the findings from Mpath and Monocle 3, showing two waves of arterial differentiation, one of which was presumably derived from vein & venous plexus VECs (EP6) (Fig. 5e). Taken together, trajectory analyses by distinct algorithms all indicated the occurrence of venous plexus arterialization (Fig. 5c; Supplementary information, Table S1).

We next explored whether our finding with respect to the development paths of two types of embryonic arterial VECs is conserved among mammals. Trajectory analysis within the integrated dataset of human and mouse by Monocle 3 also identified two predominant paths from primordial VECs (Fig. 5f). The gradual changes in arteriovenous features along the respective paths of these two waves of arterial VEC development were similar between human and mouse (Fig. 5g; Supplementary information, Fig. S8l). Specifically, mitotic nuclear division-related genes were enriched in the gene set with an expression pattern that showed gradual down-regulation along major artery VEC development in both human and mouse (Supplementary information, Fig. S8l). Taken together, it was basically conserved in mid-gestational vascular development between human and mouse regarding the two major types of arterial VEC populations and their developmental paths.

### Widespread capillary arterialization from venous-featured plexus during early expansion of intra-embryonic vasculatures

**Fig. 6:**
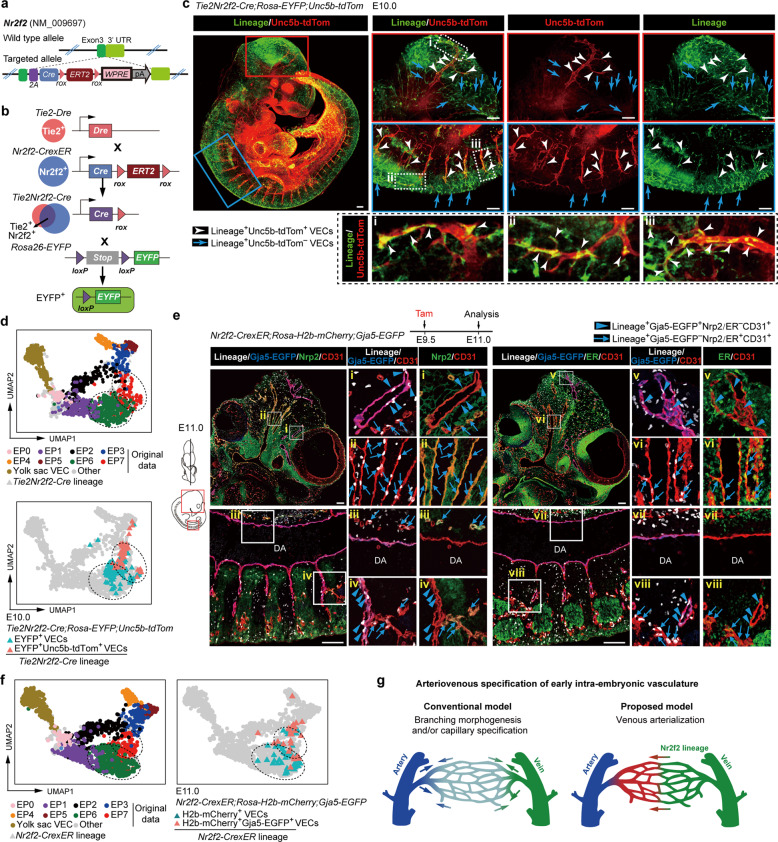
Genetic lineage tracing showing the capillary arterialization from venous-featured plexus during early expansion of intra-embryonic vasculatures. **a** Schematic model of the gene-targeting strategy for generating *Nr2f2-CrexER* mouse line via CRISPR/Cas9 system. **b** Schematic model showing the working principle for sequential Dre and Cre recombination. The final readout is Cre–loxp recombination. **c** Whole-mount immunostaining of E10.0 *Tie2Nr2f2-Cre;Rosa-EYFP;Unc5b-tdTomato* embryo with antibodies of RFP (red) and GFP (green) to detect Unc5b-tdTomato^+^ cells and *Tie2Nr2f2-Cre* lineage-traced cells, respectively. Note that in addition to the Unc5b-tdTom^−^ VECs (blue arrows), the labeling by *Tie2Nr2f2-Cre* lineage is also observed in the Unc5b-tdTom^+^ arterial VECs (white arrowheads), the distribution of which includes the branches of internal carotid arteries and the intersegmental arteries. Images in red boxes, reconstructions with a total of 70 μm thickness; images in blue boxes, reconstructions with a total of 100 μm thickness; images in dotted boxes show inserts at high magnification. Scale bars, 100 μm. **d** UMAP plots combining the cells from our original data and E10.0 *Tie2Nr2f2-Cre* lineage-labeled VECs (CD41^−^CD43^−^CD45^−^CD31^+^CD144^+^). Note that the lineage-labeled VECs are mainly clustered together with EP6 and EP7. Dashed outlines of EP6 and EP7 are indicated. **e** Representative immunostaining on sagittal sections of E11.0 *Nr2f2-CrexER;Rosa-H2b-mCherry;Gja5-EGFP* embryos after single dose of tamoxifen induction at E9.5. Note that in addition to the Gja5-EGFP^−^Nrp2/ER^+^CD31^+^ venous VECs (blue arrows), the labeling by *Nr2f2-CrexER* lineage is also observed in the Gja5-EGFP^+^Nrp2/ER^−^CD31^+^ artery plexus VECs (blue arrowheads), the distribution of which includes the branches of internal carotid arteries and the intersegmental arteries but not DA. The diagrams on the left indicate the positions of the sections and imaging. DA, dorsal aorta. Scale bars, 100 μm. **f** UMAP plots combining the cells from our original data and E11.0 *Nr2f2-CrexER* lineage-labeled VECs (CD41^−^CD43^−^CD45^−^CD31^+^CD144^+^). Note that the lineage-labeled VECs are mainly clustered together with EP6 and EP7. Dashed outlines of EP6 and EP7 are indicated. **g** Diagram showing the conventional model (left) and newly proposed model (right) regarding the arteriovenous specification of early intra-embryonic vasculature.

Finally, we explored whether arterial plexus VECs arise from arterialization of venous plexus VECs, as deduced from the bioinformatics analysis. We specifically chose the canonical venous marker Nr2f2 and generated a *Nr2f2*^*CrexER*^ mouse line which allows inducible genetic lineage tracing (Fig. 6a). First, we checked the expression of CrexER by ER immunostaining from E8.0 to E9.5 and confirmed that its expression was in general consistent with that of *Nr2f2* at the transcriptional level (Fig. 1e; Supplementary information, Fig. S9a–c). Nr2f2-CrexER was undetectable in the vasculature at E8.0, in contrast to Nrp2 that exhibited scattered expression in the aortae and ubiquitous yet inconsistent expression in other vascular primordia (Fig. 1e; Supplementary information, Fig. S9a). At later stages, Nr2f2-CrexER was expressed in the anatomically recognized veins and also in the vascular plexus, showing a mutually exclusive expression pattern with that of Dll4. This was similar to the performance of Nrp2 with regard to the vasculature (Supplementary information, Fig. S4c–e). These findings provided support for the identity of Nr2f2-CrexER as a venous-featured marker to label both early plexus VECs (EP1) and vein & venous plexus VECs (EP6) but neither primordial VECs nor arterial-featured VEC populations (Fig. 1e; Supplementary information, Fig. S9b, c). We also generated a *Tie2-Dre;Nr2f2-CrexER* mouse model, in which the Cre-mediated ablation occurred specifically in the Nr2f2-expressing VECs but not in other cell types^50^ (Fig. 6b), to constitutively trace the fate of venous-featured VECs and achieve better vasculature visualization.

Using either Rosa-EYFP or Rosa-H2b-mCherry as the reporter of Cre recombinase, we performed genetic lineage tracing. We confirmed there was no leakiness of *Nr2f2-CrexER* without tamoxifen induction (Supplementary information, Fig. S10a). At E10.0, *Tie2Nr2f2-Cre* lineage-labeled cells were widely distributed throughout the vasculature, but were seldom detected in the aorta, similar to that traced by a single dose of tamoxifen induction at E8.5 and observed at E11.0 (Supplementary information, Fig. S10b, c). These results were in line with the previous notion and also the trajectory analysis here, indicating that the initial fates of major artery VECs and venous VECs have already been segregated by the time when VECs acquire Nr2f2 expression and become venous at around E8.5 (Fig. 5b, e). Histologically, the contribution of the *Tie2Nr2f2-Cre* lineage to the Unc5b^+^ arterial plexus VECs was evident, including those located at the branches of internal carotid arteries, the intersegmental arteries, and the limb bud arterial plexus (Fig. 6c; Supplementary information, Fig. S10d). By further scRNA-seq with the isolated *Tie2Nr2f2-Cre* lineage-labeled VECs, we clearly showed the arterial characteristics of most lineage-traced Unc5b^+^ VECs with a loss of Nr2f2 expression, providing molecular validation of the conversion from venous- to arterial-featured VECs (Fig. 6d; Supplementary information, Fig. S10e, f and Table S5).

Single dose tamoxifen induction in the *Nr2f2-CrexER;Rosa-reporter* mouse model at E9.5 labeled mainly vein & venous plexus VECs (EP6) but few, if any, early plexus VECs (EP1) (Fig. 1d, e). Importantly, at E11.0, Gja5^+^ and Unc5b^+^ arterial plexus VECs but not aortic VECs were evidently lineage-traced by E9.5 induction, although the tracing efficiency varied at least in part due to the different Rosa-reporter systems (Fig. 6e; Supplementary information, Fig. S11a). Furthermore, the ER expression in the lineage-traced arterial plexus VECs was largely diminished, validating the conversion from venous- to arterial-featured VECs histologically (Fig. 6e; Supplementary information, Fig. S11a). Flow cytometric analysis further confirmed that tamoxifen treatment at E9.5 led to the labeling in both Dll4^−^CD44^−^ VECs (presumed EP6, vein & venous plexus VECs) and Dll4^+^CD44^−^ VECs (presumed EP7, arterial plexus VECs) in multiple embryonic sites, but seldom in Dll4^+^CD44^+^ VECs in the aorta-gonad-mesonephros (AGM) region (major artery VECs) (Supplementary information, Fig. S11b). We also performed additional scRNA-seq of the *Nr2f2-CrexER* lineage-labeled VECs with Gja5-EGFP as an arterial indicator, and validated the unambiguous arterial identity of partial *Nr2f2-CrexER* lineage-traced cells when induced at E9.5 (Fig. 6f; Supplementary information, Fig. S11c, d and Table S5).

Regarding the distribution of the lineage-traced cells within the vasculature and their contribution to the arterial-featured VECs, the lineage-tracing results of those induced at E9.5, those induced at E8.5, or using the constitutive *Tie2;Nr2f2-Cre* system were similar. This indicated that the conversion to an arterial fate predominantly occurred from the population with more apparent venous characteristics (EP6) rather than directly from early plexus VECs (EP1), in line with the prediction of the trajectory analysis (Fig. 5a, b, e). Taken together, genetic lineage tracing clearly revealed early and extensive capillary arterialization from venous-featured plexus during mouse embryonic development, involving multiple anatomical sites. This reshapes the conventional model of arteriogenesis, which predominantly involves branching morphogenesis from pre-existing arteries and symmetric remodeling with arterial specification from unspecialized capillaries during the expansion of early vasculature^4^ (Fig. 6g).

## Discussion

Our present data provide unprecedentedly comprehensive details of the single-cell mapping of early embryonic VECs in both human and mouse. Specifically, we revealed evolutionarily conserved evident arteriovenous features of early intra-embryonic vasculatures. Importantly, most of the in silico-identified VEC populations here were immunophenotypically and anatomically determined and further transcriptomically validated. The arterial plexus VECs identified here presumably correspond to the pre-artery cells that are defined in the coronary capillary vessels, as both of them are morphologically indistinguishable from their neighboring VECs in the vascular plexus but display apparent arterial characteristics. We also found that they share several novel arterial markers, such as *Slc45a4* (Supplementary information, Fig. S8j), *Mecom* (Fig. 4f), *Igfbp3* (Fig. 4c, f), and *Ptp4a3*^4,10^ (Fig. 1e; Supplementary information, Fig. S6e).

During mammalian development, the venous-to-arterial fate conversion is known to occur in later stages of organogenesis and be confined to specific organs; e.g., the coronary arteries are initially formed from venous VECs of the sinus venosus.^10,11^ To the best of our knowledge, the arterialization of venous-featured VECs that we revealed here represents the earliest and most extensive fate conversion from venous- to arterial-featured VECs reported to date in mammals. In addition to the fate conversion from venous-featured VECs revealed here, arterial capillaries might be formed in other potential ways, including de novo generation from angioblasts, sprouting from pre-existing arterial VECs, and arterialized from unspecialized capillary VECs.^3,21,51,52^ Except for the initial generation of preliminary vasculature from mesodermal progenitors in early embryos, such as the formation of dorsal aorta, cardinal veins, and vitelline vessels,^3,21,51^ the aortic arch arteries are reported to be derived from the second heart field mesoderm via vasculogenesis at mid-gestation.^53–55^ Whether and to what extent arterial capillaries are directly formed from angioblasts in mammalian embryos is still unclear, which needs further investigations, e.g., with the help of newly developed complicated lineage tracing systems.^56,57^ Notably, although the intersegmental arteries are proposed to be formed by endothelial sprouting from the aortas as evidenced by three-dimensional visualization of the CD31^+^ vasculature in mouse embryo,^19^ a more rigorous study using genetic lineage-tracing strategy with a *Gja5-CreER* mouse model by tamoxifen induction at E9.5 indicates that Gja5 lineage-labeled VECs are abundantly observed in aortas but seldom detected in the intersegmental arteries at later stages.^58^ Together with our findings, these data highlight the notion that capillary arterialization from venous-featured VECs rather than arterial sprouting from major artery VECs predominantly contributes to the formation of intersegmental arterial VECs. Therefore, our finding reshapes the conventional model regarding the arteriogenesis behaviors of early intra-embryonic vasculatures. The rapid acquisition of arterial characteristics directly in venous-featured VECs in the vascular plexus of the embryo proper occurs concurrently with the dramatic cellular expansion and precedes the development of major organs in the embryo,^17^ which suggests an efficient way to cooperate with organogenesis. Although embryonic arterial plexus VECs are starting to show an arterial identity on a molecular level, their future fate may not be fixed at this time point, which will depend on specific microenvironment cues.^10,12,41,43^

These findings together will shed new light on the understanding of adult arteriogenesis, especially with regard to the poorly understood process of capillary arterialization, which will have obvious diagnostic and therapeutic impacts for many important diseases and conditions.^6^ The present data also provide an invaluable transcriptomic resource for deeply investigating the mechanisms underlying arteriovenous specification of VECs, which will without doubt pave the way for studies in the vascular regeneration field.

## Materials and methods

### Mice

Mice were handled at the Laboratory Animal Center of Academy of Military Medical Sciences in accordance with institutional guidelines. Mouse manipulations were approved by the Animal Care and Use Committee of the Institute. *Gja5*^*EGFP/+*^, *Kitl*^*GFP/+*^, *Rosa*^*LoxP-Stop-LoxP-EYFP*^*, Rosa*^*LoxP-Stop-LoxP-H2b-mCherry*^ and *Tie2*^*Dre/+*^ mice were described previously.^50,59–62^ The *Dll4*^*tdTomato/+*^*, Unc5b*^*tdTomato/+*^and *Nr2f2*^*CrexER/+*^ mouse lines were generated with the CRISPR/Cas9 technique by Beijing Biocytogen and Shanghai Model Organisms Center, respectively. All mice were maintained on C57BL/6 background. Embryos were staged by somite pair (sp) counting: E8.0, 1–7 sp; E8.5, 8–12 sp; E9.0, 13–20 sp; E9.5, 21–30 sp; E10.0, 31–35 sp; E10.5, 36–40 sp; and E11.0, 41–45 sp. For well-based scRNA-seq, each embryo was isolated and further dissected into several parts including yolk sac, head, body, and limb buds, excluding heart, visceral bud, and vitelline and umbilical vessels outside embryo proper. For droplet-based scRNA-seq (10× Genomics), embryo proper was collected as a whole excluding heart, visceral bud, and vitelline and umbilical vessels outside embryo proper. In some experiments, caudal half of E10.0 embryo was dissected under heart with limbs removed. The AGM region was dissected as reported.^63^ The dorsal part of caudal half after the AGM region removed was collected as trunk. To specifically capture aortic luminal VECs, we performed microinjection of fluorescent dye Oregon Green into the dorsal aortas of E10.0–E11.0 embryos as reported.^63^ The fluorescent dye Oregon Green 488 was purchased from Invitrogen. Staining was performed as previously described^63^ except that the concentration of staining solution was 5 μmol/L and the time of staining was 3 min before washed. Primary embryonic single-cell suspension was acquired by type I collagenase digestion. For lineage tracing, tamoxifen (sigma, T5648) was dissolved in corn oil and administered to pregnant mice by oral gavage at the indicated time. Pregnant mice received 0.1 mg tamoxifen per gram body weight.

### Human embryonic sample collection and ethics statement

Human embryonic samples were acquired immediately after elective medical termination of pregnancy in Affiliated Hospital of Academy of Military Medical Sciences (the Fifth Medical Center of the PLA General Hospital). Informed consent in writing was signed before sample collection and all experiments were performed in accordance with protocols approved by the Ethics Committee of the Affiliated Hospital of Academy of Military Medical Sciences (ky-2017- 3-5). Carnegie stages (CS) were used to determine the stages of embryos according to the sp number: CS10, 8–12 sp; CS11, 13–20 sp; CS12, 21–29 sp; CS13, 30–34 sp. For flow cytometric analysis and sorting, head and limb were dissected in the same way as mouse, the rest part of embryos were collected as samples named body. Primary embryonic single-cell suspension was acquired by type I collagenase digestion.

### Flow cytometry

Cells were sorted and analyzed by flow cytometers FACS Aria 2 (BD Biosciences), and the data were analyzed using FlowJo software (Tree Star). For well-based scRNA-seq, the sampling cells were purified by FACS as CD45^−^CD31^+^CD144^+^, which contained predominantly VECs and a subset of hematopoietic cells with apparent CD41 expression. Meanwhile, CD45^−^CD31^−^CD144^−^ non-ECs in the body were used as negative controls. For droplet-based scRNA-seq (10× Genomics), only immunophenotypic VEC population (CD45^−^CD31^+^CD144^+^) were collected. Cells from mouse were stained by the following antibodies: CD31 (BD or BioLegend, MEC13.3), CD41 (BD or eBioscience, MWReg30), CD43 (BD, S7), CD44 (eBioscience or BioLegend, IM7), CD45 (eBioscience, 30-F11), CD144 (eBioscience, eBioBV13), Dll4 (BioLegend, HMD4-1), CD140b (BioLegend, APB5) and Ki67 (BD). For cell cycle analysis, cells were fixed using Fixation and Permeabilization Solution (BD) after surface marker staining, and then Ki67 and Hoechst (Sigma) staining were performed. Cells from human embryos were stained by the following antibodies: CD31 (BD, WM59), CD34 (BD, 563) and CD45 (BD, 2D1). 7-amino-actinomycin D (7-AAD; eBioscience) was used to exclude dead cells.

### Immunofluorescence

Embryos were isolated, fixed with 4% paraformaldehyde for 30 min to 2 h at 4 °C, embedded in paraffin, and sectioned at 5–6 μm with Leica RM2235. Sections were deparaffinized with ethanol of gradient concentration, then blocked in blocking solution (Zhongshan golden bridge) for 30 min at room temperature (RT), followed by incubation with primary antibodies overnight at 4 °C. After 3 washes for 3 min each in PBS, sections were incubated with corresponding secondary antibodies (Zhongshan golden bridge) for 30 min at RT. After 3 washes in PBS, sections were stained with TSA Plus Fluorescein Kit (Histova, NEON 5-color IHC Kit for FFPE, 1:100, 20–60 s). The antibodies were thoroughly eluted by heating the slides in citrate buffer (pH 6.0) for 20 min at 95 °C using microwave. After that, the next primary antibody was detected following the steps above. Each antigen was labeled by distinct fluorophores. After all the antibodies were detected sequentially, the sections were finally stained with DAPI. Images were collected by confocal microscope (Nikon Ti-E A1/ZEISS LSM 880). The primary antibodies were as follows: αSMA (Cell Signaling, 1:1000), CD36 (Abcam, 1:100), CD44 (BD Biosciences, 1:40), Estrogen Receptor α (Abcam, 1:200), Endomucin (eBioscience, 1:100), GFP (Cell Signaling, 1:200), Ltbp4 (R&D, 1:400), Nrp2 (Cell Signaling, 1:150), Pdgfrβ (Cell Signaling, 1:100), CD31 (Cell Signaling, 1:100 or Abcam, 1:1000), ERG (Abcam, 1:100), Runx1 (Abcam, 1:200), BrdU (Abcam, 1:300), and RFP (Rockland, 1:1000).

### RNAscope mRNA in situ hybridization assay combined with immunofluorescence

mRNA expression was determined using double-Z design RNA probes specifically designed to hybridize with Cyp26a1/Ptp4a3 molecules (Advanced Cell Diagnostics, Hayward, CA). RNAscope assay was performed carefully step by step according to the RNAscope® Multiple Fluorescent Reagent Kit v2 Assay (323100-USM; Advanced Cell Diagnostics, Newark, CA). Briefly, tissues were pre-treated with heat and protease prior to hybridization of the target probe. Preamplifier, amplifier and an alkaline phosphatase-labelled oligos were sequentially hybridized followed by the application of TSA Plus Fluorescein Kit (Histova, NEON 4-color IHC Kit for FFPE, 1:300, 40 °C for 20 min) to produce punctate dots. After the hybridization, the tissues were blocked with 10% normal serum in TBS-1% BSA for 30 min at RT, followed by the incubation with ERG primary antibody (Abcam) overnight at 4 °C. After 3 washes for 5 min each in TBST, sections were incubated with corresponding HRP-conjugated secondary antibody (Zhongshan golden bridge) for 30 min at RT. After 3 washes in TBST, sections were stained with TSA Plus Fluorescein Kit (Histova, NEON 4-color IHC Kit for FFPE, 1:100, 1–2 min) and finally stained with DAPI. Images were captured under a confocal fluorescence microscope (ZEISS LSM 880).

### BrdU cell proliferation assay

Mice at E10.0 of gestation were weighed and injected with 0.1 μg/g BrdU. Half an hour after injection, the mice were euthanized via cervical dislocation. Embryos were isolated immediately, fixed with 4% paraformaldehyde for 2 h at 4 °C and embedded in paraffin. BrdU was detected by treating tissue sections with 2 N HCl for 20 min at 37 °C and incubating BrdU primary antibody (Abcam, 1:100) overnight at 4 °C. After 3 washes for 2 min each in PBS, a corresponding HRP-conjugated secondary antibody (Zhongshan golden bridge) was applied for 30 min at RT. After 3 washes for 2 min each in PBS, sections were stained with TSA Plus Fluorescein Kit (Histova, NEON 5-color IHC Kit for FFPE, 1:100, 20–60 s). After all antigens were labeled, images were captured on an Akoya Vectra Polaris (PerkinElmer). The numbers of BrdU^+^ endothelial cells in each EP cluster were measured using ImageJ software from merged images.

### Retina whole-mount immunostaining

Mouse retina whole mount immunostaining was prepared as described in previously published standard protocol^64^ with minor modifications. Briefly, after anesthesia, the ocular globes were removed immediately and fixed in the ice-cold 4% paraformaldehyde for 2 h. Eyes were washed with PBS for at least 5 min, and then the retinas were dissected and cut into four radial incisions under a stereomicroscope (Leica MC170 HD). Dissected retinas were incubated and permeabilized overnight at 4 °C with blocking buffer (1% BSA, 0.5% Triton X-100). The retinas were washed and then incubated with incubation buffer (2% goat serum, 1% bovine serum albumin (BSA), and 0.5% Triton X-100 in PBS) for 1 h at RT, and stained with primary antibody in incubation buffer diluted 1:1 with PBS at 4 °C overnight. Retinas were then washed with PBS and incubated for 2 h at RT with suitable species-specific IgG (Zhongshan golden bridge) in incubation buffer (0.5% bovine serum albumin (BSA), and 0.25% Triton X-100 in PBS). After 3 washes in PBS, retinas were stained with TSA Plus Fluorescein Kit (Histova, NEON 4-color IHC Kit for FFPE, 1:100, 20–60 s). Images were captured under a confocal fluorescence microscope (Nikon Ti-E A1).

### Embryo whole-mount immunostaining

E10.0 embryos were fixed in 2% paraformaldehyde for 0.5 h on ice. Immunostaining was performed as described below. Samples were bleached in 5% H_2_O_2_ for 1 h on ice to block endogenous peroxidase. After that, samples were incubated for 1 h in blocking solution (0.2% bovine serum albumin, 1% milk, 0.4% (v/v) Triton X-100 in PBS), then incubated overnight at 4 °C with the designated primary antibodies in blocking solution. After 3 washes for 1 h each in PBS-MT (1% milk, 0.4% (v/v) Triton X-100 in PBS), samples were incubated with suitable species-specific IgG (Zhongshan golden bridge) in PBS-MT overnight at 4 °C. After 3 washes for 20 min each in PBS-T (0.4% (v/v) Triton X-100 in PBS), samples were stained with TSA Plus Fluorescein Kit (Histova, NEON 4-color IHC Kit for Wholemount/Cytometry, 1:500, 20 min). Finally, the samples were dehydrated in 100% methanol, soaked in graded concentrations of BABB (phenylcarbinol and benzyl benzoate, 1:2)/methanol (50%, 100%; 1 min each). Images were collected by a confocal microscope (Nikon Ti-E A1/ ZEISS LSM 880).

### scRNA-seq library construction

#### STRT-seq

For well-based scRNA-seq (modified STRT-seq), single cells in good condition were picked into lysis buffer by mouth pipetting. The scRNA-seq preparation procedure was based on STRT with some modifications.^23,65,66^ cDNAs were synthesized using sample-specific 25-nt oligo dT primer containing 8-nt barcode (TCAGACGTGTGCTCTTCCGATCT-XXXXXXXX-NNNNNNNN-T25, X representing sample-specific barcode whereas N standing for unique molecular identifiers (UMIs)) (Supplementary information, Table S8) and template switch oligo (TSO) primer for template switching.^67–69^ After reverse transcription and second-strand cDNA synthesis, the cDNAs were amplified by 17 cycles of PCR using ISPCR primer and 3' Anchor primer (Supplementary information, Table S8). Up to 56 samples were pooled and purified using Agencourt AMPure XP beads (Beckman). Four cycles of PCR were performed to introduce index sequence (Supplementary information, Table S8). After this step, 400 ng cDNAs were fragmented to around 300 bp by covaris S2. The cDNA was incubated with Dynabeads MyOne^TM^ Streptavidin C1 beads (Thermo Fisher) for 1 h at RT. Libraries were generated using KAPA Hyper Prep Kit (Kapa Biosystems). After adaptor ligation, the libraries were amplified by 7 cycles of PCR using QP2 primer and short universal primer (Supplementary information, Table S8). The libraries were sequenced on Illumina HiSeq 4000 platform in 150 bp paired-end manner (sequenced by Novogene and Berry Genomics).

#### 10× Genomics

For droplet-based scRNA-seq (10× Genomics), embryos from same stages with hearts removed were pooled together to acquire single-cell suspension. Cells from each stage were sorted and pooled together. Libraries were produced with a Chromium system (10× Genomics, PN120263) following the manufacture’s instruction and sequenced on Illumina Hiseq X Ten platform in 150 bp paired-end manner (sequenced by Novogene and Berry Genomics).

### Overview of generated scRNA-seq datasets

We generated multiple VEC-related scRNA-seq datasets using different technologies (modified STRT-seq and 10×), different species (mouse and human), and different mouse models. Totally, 18,970 single cells were sequenced. Specifically, by using modified STRT-seq method, we generated (i) VEC dataset (Fig. 1b, c; Supplementary information, Table S1, *n* = 2213) and mural cell dataset (Fig. 3d; Supplementary information, Table S6, *n* = 432) of mouse wild-type embryos; (ii) 4 validation datasets of 2 mouse reporter models of *Dll4-tdTom* (Fig. 2d; Supplementary information, Table S5, *n* = 288) and *Unc5b-tdTom* (Fig. 2d; Supplementary information, Table S5, *n* = 144), and 2 mouse lineage-tracing models of *Tie2Nr2f2-Cre;Rosa-EYFP;Unc5b-tdTom* (Fig. 6d; Supplementary information, Table S5, *n* = 192) and *Nr2f2-CrexER;Rosa-H2b-mCherry;Gja5-EGFP* (Fig. 6f; Supplementary information, Table S5, *n* = 112); and (iii) human embryo VEC dataset (Fig. 4a; Supplementary information, Table S7, *n* = 996). In addition, we also generated (iv) VEC 10× dataset (Supplementary information, Fig. S3 and Table S4, *n* = 10,465) of mouse wild-type embryos using 10× Genomics method.

### Processing of scRNA-seq raw data

#### STRT-seq

For modified STRT-seq data, we used UMI-based scRNA-seq method to accurately measure the gene expression profiles within individual cells. Raw reads of each cell were first split by specific barcode attached in Read 2. The corresponding Read 1 was trimmed to remove the TSO sequence and polyA tail sequence after UMI information was aligned to it. Subsequently, we conducted quality control to discard reads with adapter contaminants or low-quality bases (*N* > 10%). Next, the mm10 (for mouse data) or hg19 (for human data) transcriptome (UCSC) was used to align the clean reads through TopHat (version 2.0.12).^70^ Uniquely mapped reads were obtained by HTSeq package^71^ and grouped by the cell-specific barcodes. Duplicated transcripts of each gene were removed based on the UMI information. For each individual cell, the copy number of transcripts of a given gene was the number of the distinct UMIs of that gene. Finally, expression levels were normalized by log(TPM/10 + 1), where TPM (transcripts-per-million) was calculated as (the number of UMIs of each gene/all UMIs of a given cell) ×1,000,000. Since the UMI number of most of our samples was less than the order of 1,000,000 transcripts, the TPM values were divided by 10 to avoid counting each transcript for several times.

#### 10× Genomics

Sequencing data from 10× Genomics were processed with the Cell Ranger software (version 2.1.0) for each sample. Data were mapped to the mm10 (for mouse data) or hg19 (for human data) reference data (version 1.2.0) downloaded from 10× Genomics official website. The UMI data matrixes of quantitative gene expression for each sample were obtained. Expression levels were normalized by log(TPM/100 + 1), since the UMI number of most of our samples sequenced by using 10× Genomics method was on the order of 10,000 transcripts.

### Quality control of scRNA-seq data

#### STRT-seq

We applied slightly different but strict criteria to different datasets, taking into account the different sequencing depths. For mouse VEC and MC datasets, respectively, 2066 (93.4%) and 400 (92.6%) single cells with more than 2000 genes and 100,000 transcripts detected were retained. For 3 mouse validation datasets of *Dll4-tdTom*, *Unc5b-tdTom*, and *Tie2Nr2f2-Cre;Rosa-EYFP;Unc5b-tdTom* models, only cells with more than 2000 genes and 50,000 transcripts detected were retained. Then 274 (95.1%), 131 (91.0%) and 174 (90.6%) single cells, respectively for each dataset, passed the filter standards. For mouse validation dataset of the *Nr2f2-CrexER;Rosa-H2b-mCherry;Gja5-EGFP* model, 85 (75.9%) single cells with more than 1000 genes and 10,000 transcripts detected were retained. For human VEC dataset, 835 (86.4%) single cells with more than 2000 genes and 10,000 transcripts detected were retained.

#### 10× Genomics

Filtered cells by Cell Ranger from VEC 10× dataset of mouse wild-type embryos were retained. Data from two biological replicates were concatenated into a single dataset including 12,146 cells. We removed 1681 cells from further analyses as they clustered separately from the main group and did not express any distinct markers. Based on their expression profiles, these cells were probably low-quality cells.

### Seurat analysis procedure for scRNA-seq datasets

Seurat (version 3.1.5)^72,73^ was employed for advanced analysis of STRT-seq datasets. For different STRT-seq datasets, we followed the similar Seurat analysis procedure. Briefly, we first used ‘SCTransform’ function to perform the workflow of data normalization, identification of highly variable genes (HVGs) and data scaling, and then used ‘RunPCA’ function to perform PCA dimensionality reduction. Selected top PCs were retained using the elbow method for computing the UMAP dimensional reduction^74^ and constructing a Shared Nearest Neighbor (SNN) Graph. We used a SNN modularity optimization-based clustering algorithm, implemented in ‘FindClusters’ function, to identify clusters of cells. Parameter ‘resolution’ was used to control the number of clusters of cells.

### Analysis of VEC dataset from mouse wild-type embryos

#### STRT-seq

Following the Seurat analysis procedure described above, we initially achieved three major groups, namely VEC, Hematopoietic cell and Non-EC (Supplementary information, Fig. S2a and Table S1). Then, some cells that were not in the scope of our interest were excluded for further analysis: (i) negative control cells with a non-EC immunophenotype, (ii) cells belonging to groups of Hematopoietic cell and Non-EC, (iii) cells with *Ptprc* expression level of log(TPM/10 + 1) greater than 1, and (iv) allantoic VECs, which were identified by unsupervised clustering of all cells from E8.0–E8.5 and expression of marker genes (e.g., *Hoxa10* and *Hoxa11*). We then redid the Seurat analysis procedure to cluster the cells into yolk sac VEC group and embryo proper VEC group. Next, we further clustered the embryo proper VEC group into eight EP clusters (EP0–EP7) and the yolk sac VEC group into three YS clusters (YS1–YS3), using the Seurat analysis procedure. Notably, given the relatively limited heterogeneity and the nature of molecular continuum of the cells in EP6 and EP7, we employed a method reported previously^75^ with some modifications to optimize the clustering results between EP6 and EP7. This method combined unsupervised clustering to reveal heterogeneity in cell subtypes and supervised classification to fine-tune clusters. In brief: (i) we first identified differentially expressed genes (DEGs) between EP6 and EP7 using ‘FindAllMarkers’ function; (ii) we then performed hierarchical clustering through Pearson correlation distance metrics and got two clusters at the first split; (iii) to select feature genes dividing the two clusters, we performed a 10-fold random forest feature selection; (iv) to adjust initial classifications, we selected samples with internal vote probabilities > 0.6 for each class as the training set to achieve an optimal classifier, which was used to predict the rest of the samples; (v) we performed 100 runs of 10-fold random forest cross validation (CV) and discarded samples with internal vote probabilities < 0.55. We used internal vote probabilities > 0.55 (default = 0.5) as the cutoff to reduce the ambiguity of sample voting. For example, if the probability of assigning a sample to cluster A is 51% and to cluster B is 49%, we assume the sample is ambiguous to be classified to cluster A and need to be eliminated during classification. We used default parameters for number of trees. In this way, fine-tuned clusters of EP6 and EP7 were identified.

#### 10× Genomics

Downstream bioinformatics analyses were done with Scanpy (version 1.4.3).^76^ The data were normalized using standard total-count normalization and then taken log1p transformation as expression level. Genes with normalized dispersion > 0.5 and average expression > 0.5 but < 5 were selected as HVGs. The expression data was scaled with unit variance and zero mean. The scaled data of HVGs were used for PCA. Top 25 PCs were retained using the elbow method for computing the neighborhood graph and embedding the neighborhood graph using UMAP.^74^ Finally, Louvain algorithm^77^ was employed to cluster cells into subgroups. DEGs were identified using ‘logreg’ method.^78^

### Identification of DEGs

#### STRT-seq

We used the FindAllMarkers function and FindMarkers function in Seurat to identify cluster feature genes between multiple clusters and DEGs between two given clusters, respectively. The log(TPM/10 + 1) transformed expression values and ‘wilcox’ method were used. Only genes that are detected in a minimum fraction of 0.25 cells in either of two populations were included for test. Genes with fold-change ≥ 2 and adjusted *P* value ≤ 0.05 were retained as DEGs.

#### 10× Genomics

DEGs were identified using ‘logreg’ method^78^ implemented in Scanpy.

### GO enrichment analysis

Strict thresholds were used for identification of specific marker genes between clusters as described above in DEG analysis, which resulted in fewer genes (less than 100 DEGs in most cases) being retained. To achieve adequate number of DEGs for enrichment analysis, we tuned down the threshold of fold-change to 1.5. Then the top 200 DEGs (if any) were used for subsequent analysis. Network enrichment analysis was performed using Metascape^79^ (http://metascape.org). GO biological process enrichment analysis was performed using clusterProfiler.^80^

### GSEA/KEGG analysis

Gene Set Enrichment Analysis (GSEA) is a widely used computational analysis method that determines whether a predefined gene set shows statistically significant, concordant differences between two biological states. GSEA coupled with the gene set data of Kyoto Encyclopedia of Genes and Genomes (KEGG) was applied to explore signaling pathways changes by pairwise comparisons of two selected clusters. The same procedure was used for each comparison. Briefly, KEGG pathway gene sets were downloaded from Molecular Signatures Database (MSigDB version 6.1, http://www.broadinstitute.org/gsea/index.jsp) and getLDS function in biomaRt R package^81^ was employed to convert human genes to mouse homologous genes. Then we used the javaGSEA 4.1.0 with permutation 10,000 and Signal2Noise as metrics for ranking genes. We chose FDR < 0.1 to select the significantly altered pathways. Normalized enrichment scores (NES) of all significant pathways were used for illustration in heatmap, and NESs of insignificant pathways were set to zero for the sake of simplification. Hierarchical clustering of pathways and comparisons were performed on indicator matrix of pathway change direction, using Euclidean as distance metric and ward.D as agglomeration method. Indicator of pathway change direction was defined as 0 if pathway was insignificantly changed, otherwise, 1 if NES was positive and −1 if negative.

### Arterial and venous feature score

We selected a set of arteriovenous marker genes previously known or inferred from the artery development pattern genes, including 13 arterial genes (*Bmx, Cxcr4*, *Dll4*, *Efnb2*, *Epas1*, *Gja4*, *Gja5*, *Hey1*, *Igfbp3*, *Mecom*, *Nrp1, Unc5b* and *Vegfc*) and 3 venous genes (*Aplnr*, *Nr2f2* and *Nrp2*),^6,10,21,37^ to perform the arteriovenous feature score. First, we scaled the log_2_(TPM/10 + 1) expression values of each of the 20 marker genes to 0–10 scale among all the sample cells after quality control. Second, for each cell, we averaged the scaled values of arterial genes and venous genes, respectively. Third, the averaged values were rescaled to 0–10 scale across all the sample cells to finally achieve the arterial and venous score, respectively. For each population, the arterial and venous scores of all of the cells within the population were averaged, respectively, and the 50% confidence ellipse was also calculated to show the main distribution range. We chose score 5 as the threshold to infer the arterial or venous identity of VECs, as the distribution of individual cells was in line with the notion showing essentially no arterial/venous double-positive cells. In addition, homologous genes of the set of arteriovenous marker genes were used for this analysis of human VEC dataset.

### Fitting principal curve in PCA space

A principal curve is a smooth curve passing through the middle of a multi-dimensional dataset. Combined with PCA, principal curve may model mainly the gradual changes associated with cell differentiation or lineage relationships from single-cell gene expression data.^82^ In brief, (i) we used standard PCA to get default PC scores; (ii) we replaced default principal component scores with a sum of the top 60 gene scores because it has been proven to be less correlated with technical artefact and better correlated with cluster-specific genes;^10^ (iii) we applied the princurve R package^82^ to the top two PCs, the most informative dimensions underlying the transcriptome, for fitting a principal curve and inferring the pseudo-order of cells. We applied this workflow to infer the cell pseudo-order for all cells belonging to selected cell populations, such as clusters of EP1 and EP2, clusters of EP2 and EP3, clusters of EP6 and EP7 (Fig. 3a; Supplementary information, Fig. S6a).

### Identification of pattern genes of arterial vasculature

We presumed that VECs included in EP1 and EP2 was a continuum of arterial–venous identity, VECs in EP2 and EP3 were involved in the development of major arteries and VECs in EP6 and EP7 was a continuum of later vascular plexus. Up-regulated genes in EP2 compared to EP1 were assumed as arterial segregation genes, and up-regulated genes in EP3 compared to EP2 were assumed as arterial strengthening genes. These DEGs were identified using ‘wilcox’ method with fold change > 1.5 as threshold (Supplementary information, Table S3). For visualization of changes in gene expression across these spatially or temporally continuous VEC populations, we inferred the pseudo-order of cells involved in each continuum, using the method described in ‘Fitting principal curve in PCA space’. Such analysis was performed on VEC populations of EP1 & EP2, EP2 &EP3, and EP6 & EP7, respectively. All cells within the given clusters were included for analysis, regardless of sampling locations and stages. In the visualizations, cells are ordered by pseudo-order, and pattern genes are ordered firstly by pattern categories and then by *P* values of comparison between EP6 and EP7 using Wilcoxon rank sum test.

### Cell cycle analysis

We used CellCycleScoring function in Seurat to calculate the cell cycle phase scores and achieve cell cycle phase assignments for single cells. Cell cycle-related genes were used, including a previously defined core set of 43 G1/S and 54 G2/M genes^69,83^ (Supplementary information, Table S8).

### Putative interactions between endothelial cells and mural cells

We identified putative interactions between VECs with an arterial feature and their corresponding mural cells using the gene sets of potential receptor–ligand interactions collected from Ramilowski et al.^84^ Based on the spatiotemporal relationships, we defined three pairs of datasets, namely, E9.5 DA-derived mural cells vs cluster EP3 VECs, E10.5 DA-derived mural cells vs cluster EP5 VECs, and E9.5 limb bud-derived mural cells vs cluster EP7 VECs. We used log(TPM/10 + 1) transformed expression values to measure the potential interactions. If the expression value of a certain receptor in more than 50% cells of one cell type exceeded 1.34, and the expression value of the matched ligand in more than 50% cells of the other cell type also exceeded 1.34, we regarded this ligand–receptor pair as a potential heterologous cellular interaction between these two cell types (Supplementary information, Table S6). The expression threshold of 1.34 is determined by the upper quartile of expression values of all genes in the analyzed data.

### Constructing single cell trajectories

To infer the developing trajectory of individual cells or cell populations, we adopted 3 methods, namely Mpath,^48^ monocle 3^17^ and scVelo (version 0.2.1)^85^ to ensure the accuracy of trajectory analyses. For Mpath analysis, the HVGs identified using routine analysis procedure described above, were used. The cluster labels produced by our clustering procedures were used as landmark cluster assignment of individual cells, and other parameters used the default values (see https://github.com/JinmiaoChenLab/Mpath). For scVelo analysis, we used the steady-state model, as being used in velocyto^49^ to estimate RNA velocities of single cells by distinguishing unspliced and spliced mRNAs. We used velocyto command line interface to generate spliced/unspliced expression matrices and scVelo package to estimate velocity following the tutorial (https://scvelo.readthedocs.io/VelocityBasics.html). Then, we visualized RNA velocity vector field of the most fine-grained resolution on the UMAP embedding space generated by Seurat analysis. For Monocle 3 analysis, we also used the UMAP embeddings by Seurat to learn the trajectory graph and order the cells in pseudotime following the tutorial (https://cole-trapnell-lab.github.io/monocle3/docs/trajectories/). We used order_cells function to achieve the branch assignments and pseudotime values of cells by manually specifying the root nodes and end nodes in the principal trajectory graph. The root node was determined based on the sampling stage and results from other trajectory analyses.

### Integration analysis using fast-Mutual Nearest Neighbors (fastMNN)

We employed the fastMNN correction algorithm^86^ to mitigate batch effects (e.g., from different mouse models or different species) and perform data integration analysis for validation datasets or human datasets. Briefly, we used RunFastMNN function of SeuratWrappers R package to integrate datasets derived from different species, genotypes and/or library construction strategies. Then selected top dimensions in MNN space were retained for computing the UMAP dimensional reductions. Specifically, for integration of mouse VEC dataset and each validation dataset (Figs. 2d, e and 6d, f), HVGs identified in mouse VEC dataset were used as inputs.

### Analysis of VEC dataset from human embryos

We performed the same Seurat analysis procedure as described above, except that the batch effects and cell cycle phase scores of S.Score and G2M.Score were regressed out using the SCTransform function. Because both batch effects and cell cycle effects dominantly affect the dimension reduction results of human VEC dataset, which is different from the scene of mouse datasets. Then we achieved seven clusters of VEC1, VEC2, GATA4^+^HAND2^+^, GATA4^+^OIT3^+^, Hemogenic endothelial cell, Hematopoietic cell and Non-EC (Supplementary information, Fig. S8d and Table S7). Clusters of VEC1 and VEC2 were retained as VEC populations for further analysis.

Given the limited number of early VECs in human dataset, due to scarcity of human earlier embryos, we took mouse VEC dataset as reference for integration analysis to better understand conservation of VEC development in mammals. We integrated human VECs with mouse VECs in embryo proper VEC group by using the fastMNN integration analysis procedure. Specifically, mouse genes were converted into human homologous genes for combining the datasets from different species by using getLDS function in biomaRt R package.^81^ Considering that the cell cycle effects dominantly affect the dimension reduction results, we first select HVGs using SelectIntegrationFeatures function, and then excluded the genes highly correlated with cell cycle scores (absolute correlation coefficient > 0.3). HVGs after excluding putative cell cycle genes were used for the integration procedure, which largely moderated the impact of cell cycle effects on dimension analysis. In the integrated MNN space, we classified human VECs into the seven VEC clusters (EP0–EP3 and EP5–EP7) of mouse VEC reference dataset, by using k-Nearest Neighbour classification method.

We also used the same fastMNN analysis procedure described above to integrate VECs derived from three datasets of human VEC dataset generated by STRT-seq and mouse VEC datasets generated by both 10× Genomics and STRT-seq.

### Conversed genes between two species during vascular development

We focused on the two types of arterial VEC populations for further analysis. We renamed the human EP3 and EP5 combination as arterial VEC1 (hAEC1), EP7 as hAEC2, and EP6 as venous VEC (hVeEC), corresponding to the mouse EP3 and EP5 combination (major artery VECs, mAEC1), EP7 (arterial plexus VECs, mAEC2), and EP6 (vein & venous plexus VECs, mVeEC), respectively. We determined the conserved genes as shared DEGs in each cluster pair between two species. For mouse dataset, getLDS function in biomaRt R package^81^ was employed to convert mouse genes to human homologous genes. We also made an effort to identify conserved arterial genes, which were defined as those highly expressed in both arterial VEC populations (hAEC1/mAEC1 and hAEC2/mAEC2), when respectively compared with the corresponding venous VEC population hVeEC/mVeEC. We used Wilcoxon rank sum test to calculate the significance of each gene, and only genes with fold change > 2 and adjusted *P* value < 0.05 were retained as DEGs. Fisher’s method was employed to combine two or multiple independent *P* values into a single combined *P* value.

### Dynamically changed genes along arterial VEC development in both human and mouse

We applied Monocle 3 in the integrated UMAP space composed of human and mouse VECs, and achieved trajectories of two types of arterial VEC development for human and mouse simultaneously. Then, identification of pattern genes changed along trajectories were performed for human and mouse VECs, respectively. Briefly, pattern genes changed along each development path were identified by using differentialGeneTest function in Monocle 2.^87^ Top 500 significantly changed genes were retained for visualization in heatmaps for each development path. Pattern genes were clustered using kmeans method, and the number of clusters were determined by manually checking the heatmap results from larger to smaller number of clusters. Then pattern diagrams were illustrated, and enriched GO terms of genes for different patterns were calculated.

### Transcription factors

Transcription factors used in this study including 1691 human transcription factors and 1485 mouse transcription factors were download from Animal TFDB (http://bioinfo.life.hust.edu.cn/AnimalTFDB/)^88^ (Supplementary information, Table S8).

### Statistical analysis

All statistical analyses were conducted in R language. Two-sample Wilcoxon Rank Sum test was employed for comparisons of gene numbers, transcript counts, gene expression levels and feature scores between two given clusters of cells. More detailed information of statistical analysis is described above.

## Supplementary information

Supplementary information, Fig. S1

Supplementary information, Fig. S2

Supplementary information, Fig. S3

Supplementary information, Fig. S4

Supplementary information, Fig. S5

Supplementary information, Fig. S6

Supplementary information, Fig. S7

Supplementary information, Fig. S8

Supplementary information, Fig. S9

Supplementary information, Fig. S10

Supplementary information, Fig. S11

Supplementary information, Table S1

Supplementary information, Table S2

Supplementary information, Table S3

Supplementary information, Table S4

Supplementary information, Table S5

Supplementary information, Table S6

Supplementary information, Table S7

Supplementary information, Table S8

## Data Availability

All of the scRNA-seq data have been deposited in Gene Expression Omnibus under the accession number GSE94877.

## Code availability

All data were analyzed with standard programs and packages, as detailed above. Code is available on request.
