# Supplementary material for: Heterogeneity in endothelial cells and widespread venous arterialization during early vascular development in mammals
Source: Cell Res. 2022 Jan 25;32(4):333–48. doi: 10.1038/s41422-022-00615-z (PMC8975889; doi:10.1038/s41422-022-00615-z)
Supplement: Supplementary file 1 — Supplementary information, Fig. S1 [file 41422_2022_615_MOESM1_ESM.pdf]

Fig. S1

a

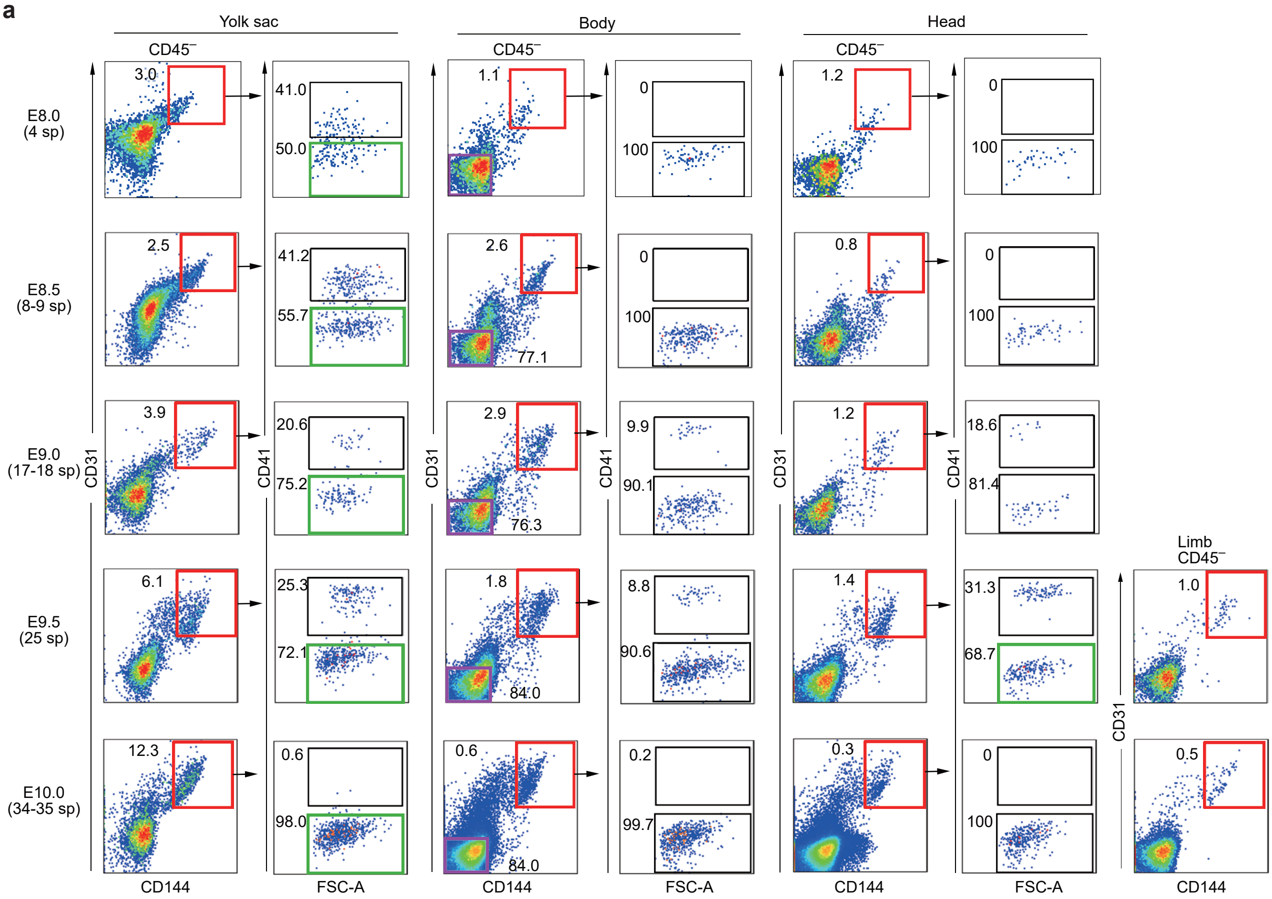

b

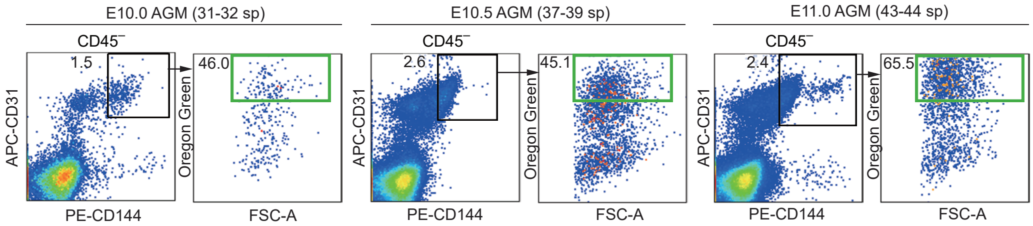

c

Stage	Embryos	Exp (n)	Cell numbers						
			YS[a+b]	Body[a]	Head[a+b]	Limb[a]	Dorsal aorta[c]	Neg in body[a]	Total
E8.0 (3-5 sp)	4	1[b]	40	40	13	/	/	8	101
E8.5 (8-9 sp)	19	3[a]+1[b]	75 + 60	236	69	/	/	50	490
E9.0 (17-18 sp)	8	2[a]+1[b]	60 + 60	205	95	/	/	40	460
E9.5 (25 sp)	6	2[a]+1[b]	60 + 60	180	90 + 40	30	/	40	500
E10.0 (31-35 sp)	12	2[a]+1[b]+2[c]	60 + 40	180	90	30	110	40	550
E10.5 (37-39 sp)	8	1[c]	/	/	/	/	56	/	56
E11.0 (43-44 sp)	7	1[c]	/	/	/	/	56	/	56
Total	64	18	274 + 241	841	357 + 40	60	222	178	2213

[a] Independent experiments for collecting sample cells (red boxes) and negative control cells (purple boxes) from different embryonic parts as shown in panel (**a**) of Fig.S1;
[b] Independent experiments for collecting $CD45^-CD31^+CD144^+CD41^-$ sample cells (green boxes) from different embryonic parts as shown in panel (**a**) of Fig.S1;
[c] Independent experiments for collecting $CD45^-CD31^+CD144^+$Oregon Green$^+$ sample cells (green boxes) from AGM region as shown in panel (**b**) of Fig.S1.

d

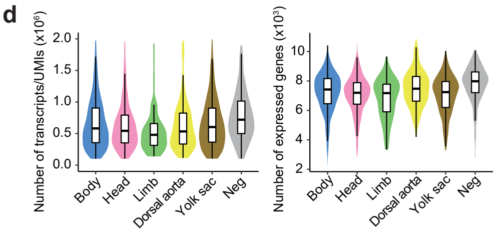

e

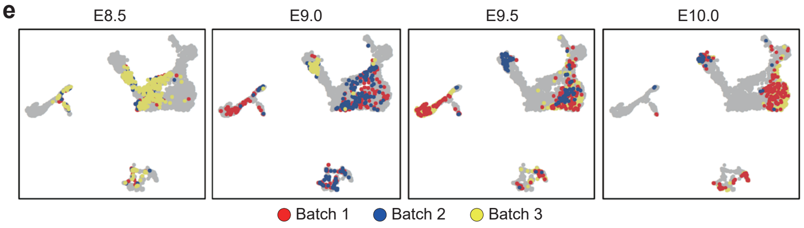

Fig. S1. Cell preparation of scRNA-seq and quality control of dataset.

a, Representative FACS plots for cell sorting. Single cell suspensions were prepared from different embryonic parts at different developmental stages as indicated. Cell populations isolated for scRNA-seq are denoted as colored boxes (red for $CD45^{-}CD31^{+}CD144^{+}$ sample cells, green for $CD45^{-}CD31^{+}CD144^{+}CD41^{-}$ sample cells, and purple for $CD45^{-}CD31^{-}CD144^{-}$ negative control cells derived from the body).
b, Representative FACS plots for sorting of aortic luminal endothelial cells. Aortic luminal layer was labeled by Oregon Green microinjection and then aorta-gonad-mesonephros (AGM) regions at different developmental stages were dissected. Green boxes indicate $CD45^{-}$ $CD31^{+}CD144^{+}$Oregon $Green^{+}$ sample cells for scRNA-seq.
c, Information for scRNA-seq, including developmental stages, numbers of embryos and independent experiments, and sampled cell numbers. YS, yolk sac; DA, dorsal aortic luminal layer of AGM region; Neg in body, negative control ($CD45^{-}CD31^{-}CD144^{-}$) cells derived from the body part. sp, somite pairs.
d, Violin plots showing the number of transcripts/UMIs and expressed genes in each of the single cells of different locations. Neg, negative control (non-endothelial) cells derived from the body part.
e, UMAP plots showing well-mixed sampling cells from different batches and no clustering bias related to experimental repetitions, indicative of no batch effect. Different batches of cells from each developmental stage are exhibited with different colors.
