# Supplementary material for: Heterogeneity in endothelial cells and widespread venous arterialization during early vascular development in mammals
Source: Cell Res. 2022 Jan 25;32(4):333–48. doi: 10.1038/s41422-022-00615-z (PMC8975889; doi:10.1038/s41422-022-00615-z)
Supplement: Supplementary file 2 — Supplementary information, Fig. S2 [file 41422_2022_615_MOESM2_ESM.pdf]

Fig. S2

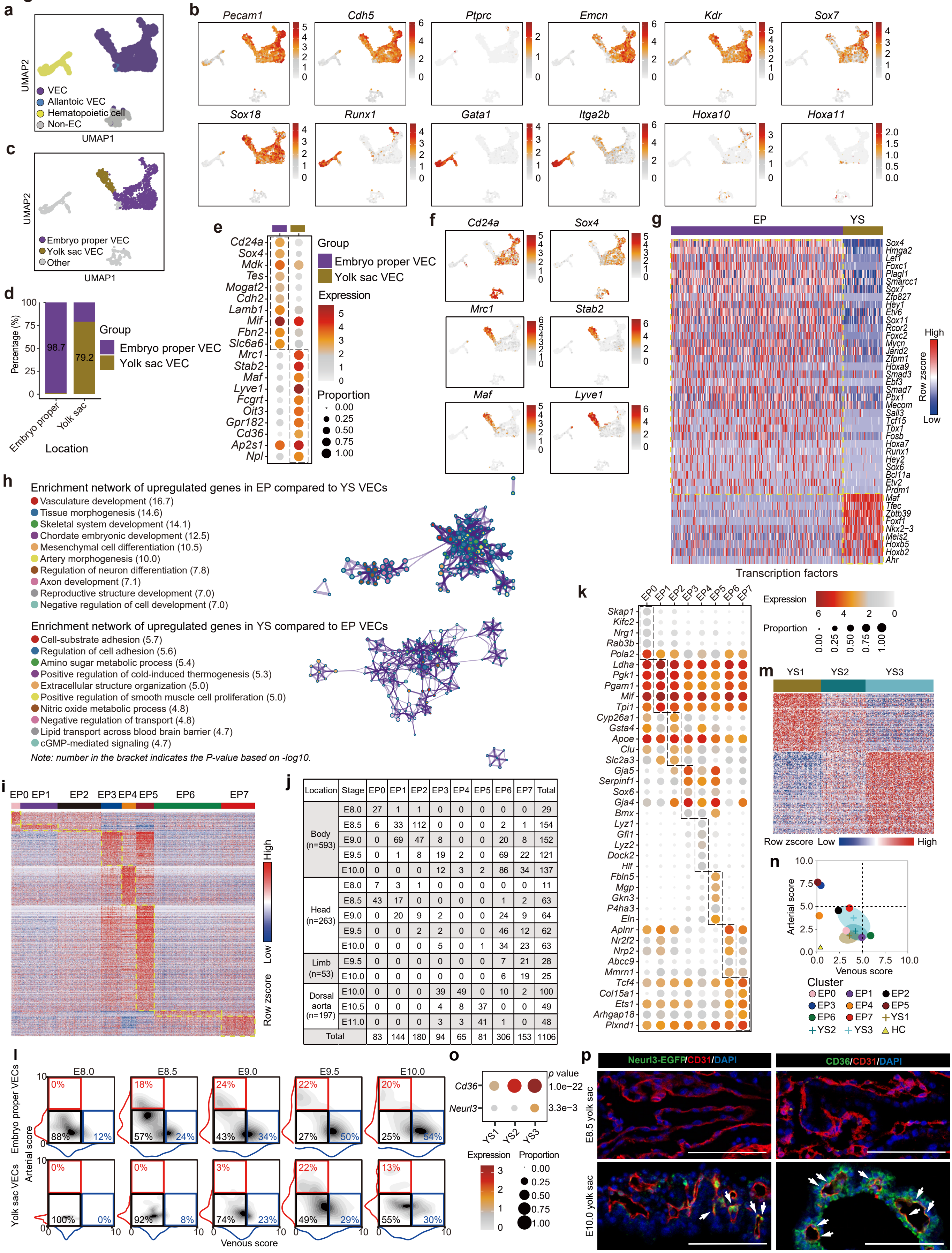

Location	Stage	EP0	EP1	EP2	EP3	EP4	EP5	EP6	EP7	Total
Body (n=593)	E8.0	27	1	1	0	0	0	0	0	29
	E8.5	6	33	112	0	0	0	2	1	154
	E9.0	0	69	47	8	0	0	20	8	152
	E9.5	0	1	8	19	2	0	69	22	121
	E10.0	0	0	0	12	3	2	86	34	137
Head (n=263)	E8.0	7	3	1	0	0	0	0	0	11
	E8.5	43	17	0	0	0	0	1	2	63
	E9.0	0	20	9	2	0	0	24	9	64
	E9.5	0	0	2	2	0	0	46	12	62
	E10.0	0	0	0	5	0	1	34	23	63
Limb (n=53)	E9.5	0	0	0	0	0	0	7	21	28
	E10.0	0	0	0	0	0	0	6	19	25
Dorsal aorta (n=197)	E10.0	0	0	0	39	49	0	10	2	100
	E10.5	0	0	0	4	8	37	0	0	49
	E11.0	0	0	0	3	3	41	1	0	48
Total		83	144	180	94	65	81	306	153	1106

Fig. S2. Molecular features of VECs derived from embryo proper and yolk sac.

a, UMAP plot of all sampled VECs and negative control cells, with four main groups mapped to it. Note that the Non-EC group contains mainly negative control cells ($CD45^{-}CD31^{-}CD144^{-}$), and three other groups comprise almost all FACS-isolated sample cells ($CD45^{-}CD31^{+}CD144^{+}$).

b, Expression patterns of indicated marker genes exhibited on UMAP plots. Notice that the cells assigned into Non-EC group largely lacked the transcripts of endothelial marker genes including *Pecam1*, *Cdh5*, *Emcn*, *Kdr*, *Sox7* and *Sox18*.

c, UMAP plot with embryo proper and yolk sac VECs mapped onto it.

d, Bar charts showing the proportion of embryo proper and yolk sac VEC groups, identified by unsupervised clustering, in different sampling sites of embryo proper and yolk sac.

e, Dot plot showing the average expression and proportion of top ten DEGs of embryo proper and yolk sac VEC groups.

f, UMAP plots showing the representative marker genes of embryo proper VECs and yolk sac VECs.

g, Heatmap showing the differentially expressed transcription factors between embryo proper VECs and yolk sac VECs.

h, Metascape network enrichment analysis with top ten enriched terms exhibited to the left. Each cluster is represented by different colors and each enriched term is represented by a circle node. Number in the bracket indicates the *P* value based on -log10.

i, Heatmap showing the relative expression levels of significantly up-regulated genes in each embryo proper VEC cluster as compared to other embryo proper VEC clusters.

j, Cell number information of the spatiotemporal distribution of eight embryo proper VEC clusters.

k, Dot plot showing the average expression levels and cell expressing proportions of top five DEGs for each embryo proper VEC cluster. Dot color indicates the average expression level within a cluster and dot size represents the number of genes within a cluster.

l, Two-dimensional density plots showing the distributions of cells in arterial/venous score maps, for embryo proper VECs (upper) and yolk sac VECs (lower), separately, at each interrogated developmental stage. The one-dimensional marginal distributions of arterial (red) and venous (blue) scores are also indicated. Zones and proportions of arterial-featured VECs, venous-featured VECs, and unspecified VECs are indicated in red, blue and black, respectively.

m, Heatmap showing the relative expression levels of significantly up-regulated genes in each yolk sac VEC cluster as compared to other yolk sac VEC clusters.

n, Scatterplot showing the average arteriovenous scores of the cells in each cluster. Main distribution ranges of arteriovenous scores in each of the three yolk sac VEC clusters are also indicated as an oval shape.

o, Dot plot showing the average and proportion expression of the selected genes in distinct yolk sac VEC clusters. The size of the dot indicates the proportion of expressing cells within a cluster, and the color indicates the average expression level of cells within a cluster. Statistics is performed by two-sided wilcoxon test between the expression in YS3 and in the other two clusters.

p, Representative immunostaining on yolk sac sections at different developmental stages as indicated. Note the E10.0 yolk sac VECs show higher expression of Neurl3-EGFP and CD36 than that of E8.5. Scale bars, 100 μm.
