# Supplementary material for: Heterogeneity in endothelial cells and widespread venous arterialization during early vascular development in mammals
Source: Cell Res. 2022 Jan 25;32(4):333–48. doi: 10.1038/s41422-022-00615-z (PMC8975889; doi:10.1038/s41422-022-00615-z)
Supplement: Supplementary file 3 — Supplementary information, Fig. S3 [file 41422_2022_615_MOESM3_ESM.pdf]

Fig. S3

a

Batch	Stage	Embryos	Proportion (%)	Cell number
1	E9.0 (15-19 sp)	9	33.3	5901
	E9.5 (22-25 sp)	10	33.3	
	E10.0 (32-34 sp)	11	33.3	
2	E9.0 (13-18 sp)	7	14.3	4564
	E9.5 (26 sp)	2	28.6	
	E10.0 (32-34 sp)	5	57.1	
Total		44	/	10465

Sorting marker: CD45⁻CD31⁺CD144⁺

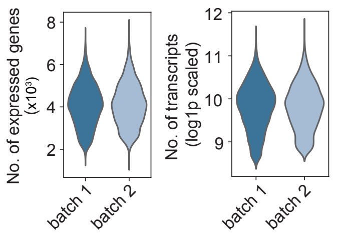

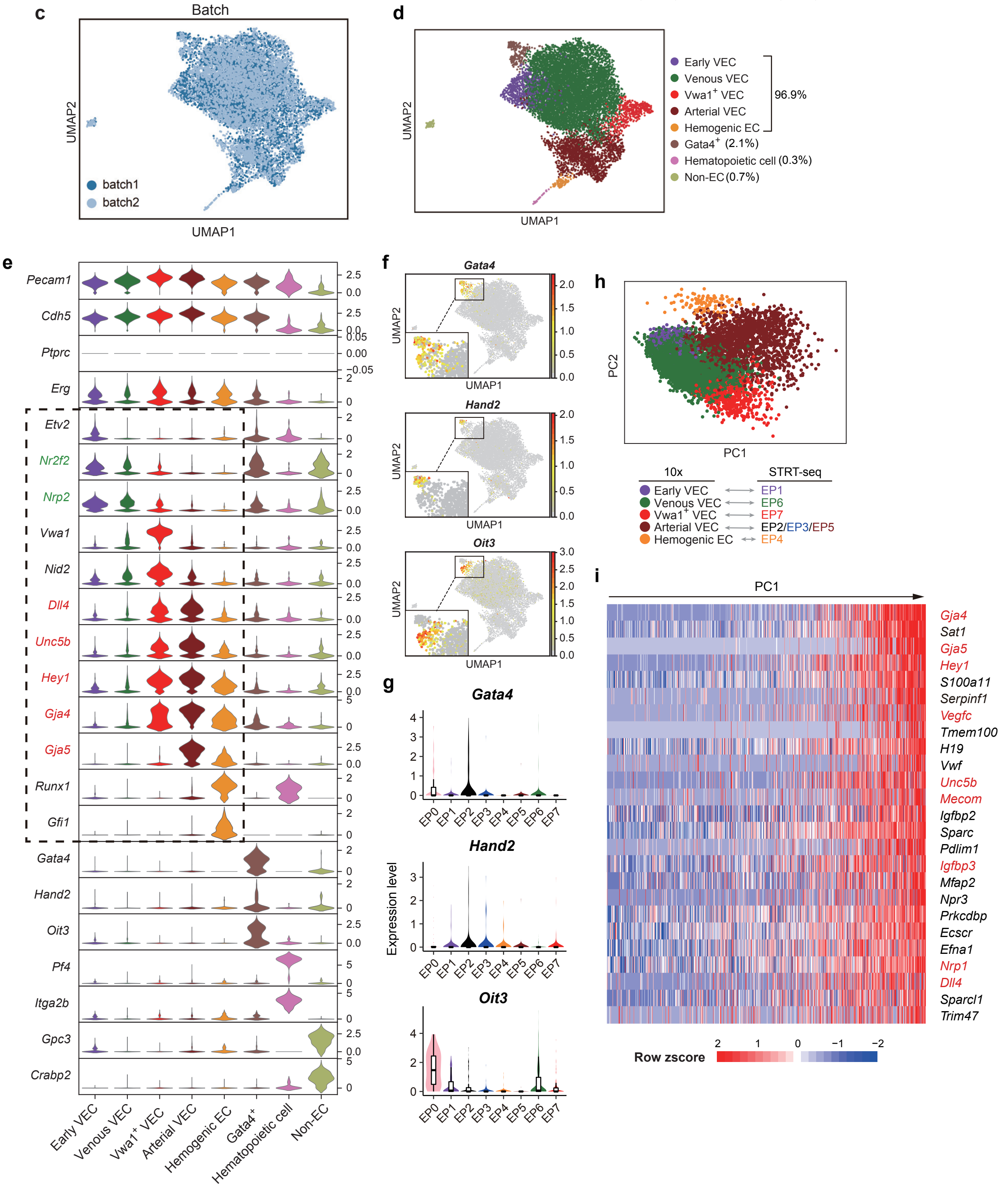

Fig. S3. Validation of principal VEC populations using alternative sequencing strategy.

a, Embryo and cell information of two biological replicates for 10x Genomics scRNA-seq. VECs were collected from embryos proper and purified by FACS.
b, Violin plots showing the number of genes (left) and transcripts (right) in each single cell of two different replicates from 10x Genomics scRNA-seq data.
c, UMAP plot showing cells from two biological replicates are evenly merged well, and no batch effect is detected from 10x Genomics scRNA-seq data.
d, UMAP plot with eight clusters from 10x Genomics scRNA-seq data identified by unsupervised clustering with louvain algorithm mapped on it. The constitutions of different populations are shown.
e, Violin plots showing expression levels of sorting markers and representative feature genes of eight clusters from 10x Genomics scRNA-seq data. Known arterial and venous genes are indicated in red and green, respectively. The dotted box indicates the VEC clusters within the scope of our concern.
f, UMAP plot from 10x Genomics scRNA-seq data with the expression levels of selected genes mapped onto it, showing that Gata4$^+$ cluster could be subdivided into two sub-clusters which likely involved cardiac (Hand2$^+$) and liver (Oit3$^+$) VECs.
g, Violin plots showing the expression of selected genes in the eight embryo proper VEC clusters identified in the well-based RNA-sequencing dataset. Note seldom Gata4$^+$ expression in any clusters.
h, PCA plot of five VEC clusters within our concern from 10x Genomics scRNA-seq data. Corresponding populations in the STRT-seq dataset are indicated to the right.
i, Heatmap showing the expression of top 25 PC1 genes along PC1 axis from 10x Genomics scRNA-seq data. Known arterial genes are indicated in red validating the arterial properties of the arterial VEC and Vwa1$^+$ VEC clusters.
