# Supplementary material for: Heterogeneity in endothelial cells and widespread venous arterialization during early vascular development in mammals
Source: Cell Res. 2022 Jan 25;32(4):333–48. doi: 10.1038/s41422-022-00615-z (PMC8975889; doi:10.1038/s41422-022-00615-z)
Supplement: Supplementary file 4 — Supplementary information, Fig. S4 [file 41422_2022_615_MOESM4_ESM.pdf]

Fig. S4

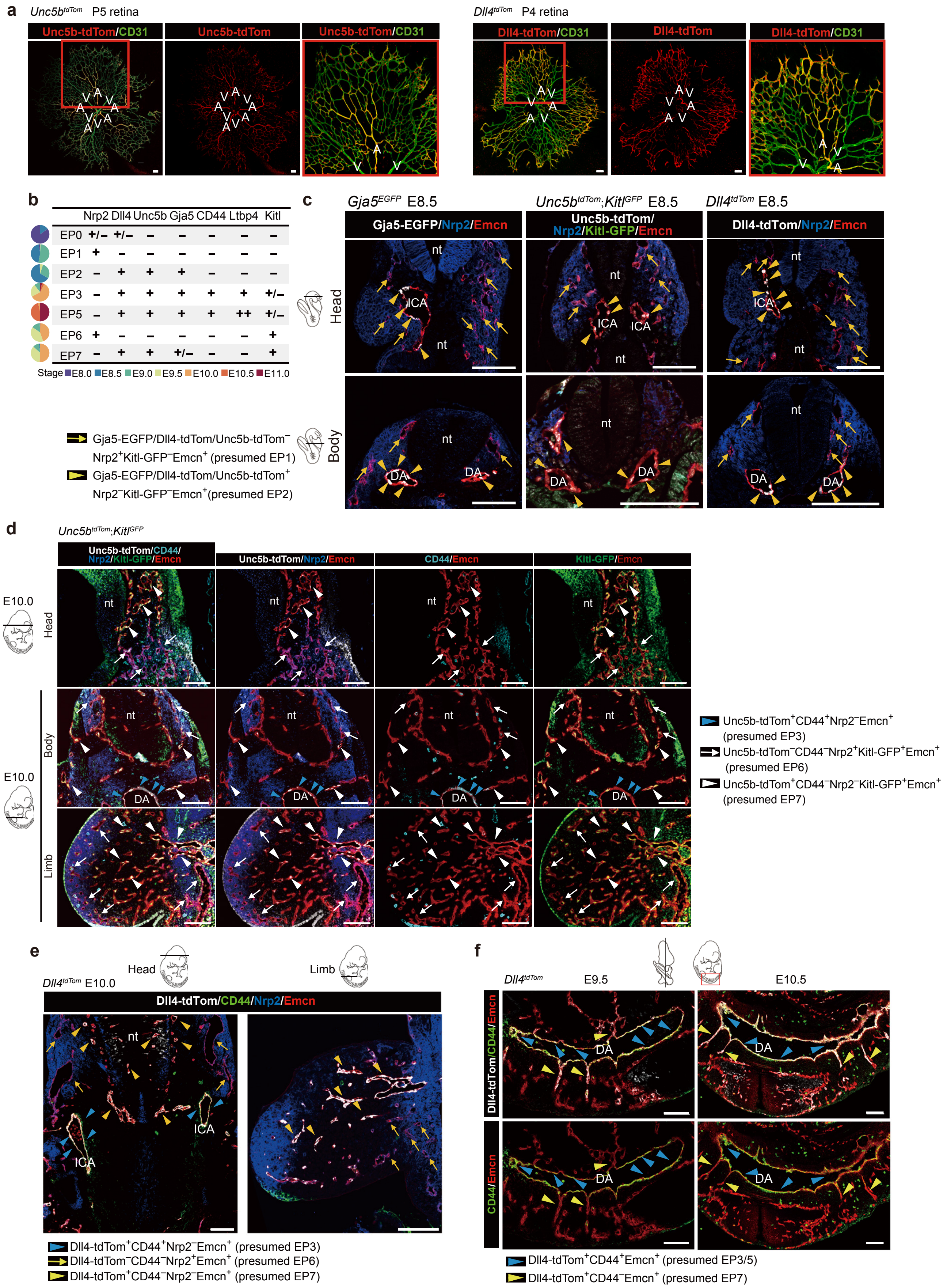
a
Unc5btdTom P5 retina
Unc5b-tdTom/CD31
Unc5b-tdTom
Dll4tdTom P4 retina
Dll4-tdTom/CD31
Dll4-tdTom
b
Nrp2 Dll4 Unc5b Gja5 CD44 Ltbp4 Kitl
EP0 +/– +/– – – – – –
EP1 + – – – – – –
EP2 – + + + – – –
EP3 – + + + + + +/–
EP5 – + + + + ++ +/–
EP6 + – – – – – +
EP7 – + + +/– – – +
Stage E8.0 E8.5 E9.0 E9.5 E10.0 E10.5 E11.0
Gja5-EGFP/Dll4-tdTom/Unc5b-tdTom– Nrp2+Kitl-GFP–Emcn+ (presumed EP1)
Gja5-EGFP/Dll4-tdTom/Unc5b-tdTom+ Nrp2–Kitl-GFP–Emcn+(presumed EP2)
c
Gja5EGFP E8.5
Unc5btdTom;KitlGFP E8.5
Dll4tdTom E8.5
Gja5-EGFP/Nrp2/Emcn
Unc5b-tdTom/ Nrp2/Kitl-GFP/Emcn
Dll4-tdTom/Nrp2/Emcn
Head
Body
nt
ICA
DA
d
Unc5btdTom;KitlGFP
Unc5b-tdTom/CD44/ Nrp2/Kitl-GFP/Emcn
Unc5b-tdTom/Nrp2/Emcn
CD44/Emcn
Kitl-GFP/Emcn
E10.0
Head
Body
Limb
Unc5b-tdTom+CD44+Nrp2–Emcn+ (presumed EP3)
Unc5b-tdTom–CD44–Nrp2+Kitl-GFP+Emcn+ (presumed EP6)
Unc5b-tdTom+CD44–Nrp2–Kitl-GFP+Emcn+ (presumed EP7)
e
Dll4tdTom E10.0
Head
Limb
Dll4-tdTom/CD44/Nrp2/Emcn
Dll4-tdTom+CD44+Nrp2–Emcn+ (presumed EP3)
Dll4-tdTom–CD44–Nrp2+Emcn+ (presumed EP6)
Dll4-tdTom+CD44–Nrp2–Emcn+ (presumed EP7)
f
Dll4tdTom
E9.5
E10.5
Dll4-tdTom/CD44/Emcn
CD44/Emcn
DA
Dll4-tdTom+CD44+Emcn+ (presumed EP3/5)
Dll4-tdTom+CD44–Emcn+ (presumed EP7)

Fig. S4. Anatomical localization of distinct vascular VEC populations.

a, Confocal images of RFP (red) and CD31 (green) double immunostaining on the postnatal day 5 (P5) *Unc5b-tdTomato* (left) and P4 *Dll4-tdTomato* (right) mouse retinas. Representative images with higher magnification are shown to the right. Note that Dll4-tdTom was clearly expressed in the arteries and their branches and also in the vascular front and Unc5b-tdTom was mainly confined to the arteries. A, artery; V, vein. Scale bars, 100 μm.
b, Table showing the expression indicator of key feature genes used in immunostaining for distinguishing seven VEC clusters. The Venn diagrams to the left represent the distribution of developmental stages for each of the embryo proper VEC cluster.
c, Representative immunostaining on cross sections of E8.5 *Gja5-EGFP* (left), *Unc5b-tdTomato;Kitl-GFP* (middle) and *Dll4-tdTomato* (right) embryos at head and body part. Arrows indicate the presumed EP1 (Gja5-EGFP/Dll4-tdTom^{-}Nrp2^{+}Emcn^{+} or Unc5b-tdTom^{-}Nrp2^{+}Kitl-GFP^{-}Emcn^{+}) located at vascular plexus. Arrowheads indicate the presumed EP2 (Gja5-EGFP/Dll4-tdom^{+}Nrp2^{-}Emcn^{+} or Unc5b-tdTom^{+}Nrp2^{-}Kitl-GFP^{-}Emcn^{+}) exclusively located at major arteries including DAs and ICAs at E8.5. The expression of Kitl-GFP is undetectable in the VECs at this early stage. The diagram to the left indicates the positions of sections. nt, neural tube; DA, dorsal aorta; ICA, internal carotid artery. Scale bars, 100 μm.
d, Representative immunostaining on cross sections of E10.0 *Unc5b-tdTomato;Kitl-GFP* embryos. Blue arrowheads indicate the presumed EP3, white arrows indicate the presumed EP6, and white arrowheads indicate the presumed EP7. Note the localization of EP3 (Unc5b-tdTom^{+}CD44^{+}Nrp2^{-}Emcn^{+}, blue arrowheads) at known arteries such as DA, and EP7 (Dll4-tdTom^{+}CD44^{-}Nrp2^{-}Emcn^{+}, white arrowheads) at vascular plexus adjacent to EP6 (Unc5b-tdTom^{-}CD44^{-}Nrp2^{+}Kitl-GFP^{+}Emcn^{+}, white arrows). EP6 and EP7 were widely distributed throughout the whole embryo, including the perineural vascular plexus, vascular sprouts within the neural tube, and capillaries in the limb buds. The diagrams to the left indicate the positions of sections. nt, neural tube; DA, dorsal aorta; Scale bars, 100 μm.
e, Representative immunostaining on cross sections of E10.0 *Dll4-tdTomato* embryos at head (left) and limb (right) regions. Note the localization of major artery VECs (Dll4-tdTom^{+}CD44^{+}Nrp2^{-}Emcn^{+}, blue arrowheads) at known arteries such as ICA, EP6 VECs (Dll4-tdTom^{-}CD44^{-}Nrp2^{+}Emcn^{+}, yellow arrows) at vascular plexus, and EP7 VECs (Dll4-tdTom^{+}CD44^{-}Nrp2^{-}Emcn^{+}, yellow arrowheads) at vascular plexus adjacent to EP6. The diagram above indicates the position of sections. nt, neural tube; ICA, internal carotid artery. Scale bars, 100 μm.
f, Representative immunostaining on sagittal sections at E9.5 and E10.5 aortic region of *Dll4-tdTomato* embryos. Blue arrowheads indicate major artery VECs (Dll4-tdTom^{+}CD44^{+} or Dll4-tdTom^{+}CD44^{+}Nrp2^{-}) located at DAs. Yellow arrowheads indicate artery plexus VECs (Dll4-tdTom^{+}CD44^{-} or Dll4-tdTom^{+}CD44^{-}Nrp2^{-}) as the extension of DAs. The diagrams above indicate the positions of sections and imaging. DA, dorsal aorta. Scale bars, 100 μm.
