# Supplementary material for: Heterogeneity in endothelial cells and widespread venous arterialization during early vascular development in mammals
Source: Cell Res. 2022 Jan 25;32(4):333–48. doi: 10.1038/s41422-022-00615-z (PMC8975889; doi:10.1038/s41422-022-00615-z)
Supplement: Supplementary file 5 — Supplementary information, Fig. S5 [file 41422_2022_615_MOESM5_ESM.pdf]

## Fig. S5

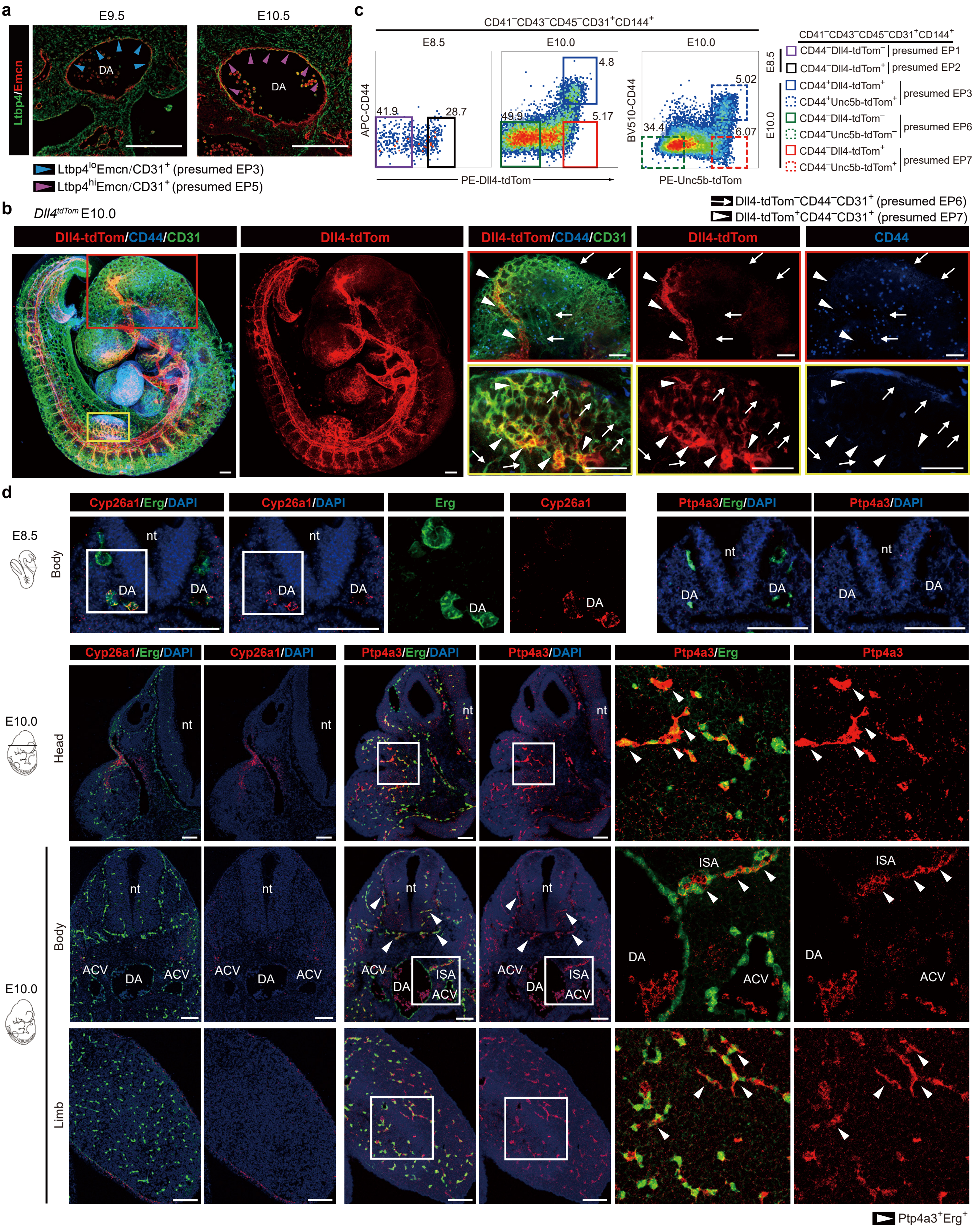

a
E9.5
E10.5
Ltbp4/Emcn
DA
Ltbp4loEmcn/CD31+ (presumed EP3)
Ltbp4hiEmcn/CD31+ (presumed EP5)
c
CD41−CD43−CD45−CD31+CD144+
E8.5
E10.0
E10.0
APC-CD44
41.9
28.7
49.9
4.8
5.17
BV510-CD44
5.02
34.4
6.07
PE-Dll4-tdTom
PE-Unc5b-tdTom
CD41−CD43−CD45−CD31+CD144+
CD44−Dll4-tdTom− | presumed EP1
CD44−Dll4-tdTom+ | presumed EP2
CD44+Dll4-tdTom+
CD44+Unc5b-tdTom+ | presumed EP3
CD44−Dll4-tdTom−
CD44−Unc5b-tdTom− | presumed EP6
CD44−Dll4-tdTom+
CD44−Unc5b-tdTom+ | presumed EP7
b
Dll4tdTom E10.0
Dll4-tdTom−CD44−CD31+ (presumed EP6)
Dll4-tdTom+CD44−CD31+ (presumed EP7)
Dll4-tdTom/CD44/CD31
Dll4-tdTom
CD44
d
E8.5
Body
Cyp26a1/Erg/DAPI
Cyp26a1/DAPI
Erg
Cyp26a1
Ptp4a3/Erg/DAPI
Ptp4a3/DAPI
nt
DA
E10.0
Head
Body
Limb
Ptp4a3/Erg
Ptp4a3
ACV
ISA
Ptp4a3+Erg+

**Fig. S5. Identification of distinct VEC populations by whole-mount immunostaining and RNAscope.**

**a,** Representative immunostaining on cross sections of aortic region of mouse embryos at different developmental stages as indicated. Note the inner layer of E10.5 and E11.0 aortas (purple arrowheads) show higher $Ltbp4^{+}$ fluorescent signals than that of E9.5 (blue arrowheads), indicative of its mature arterial VEC characteristics. DA, dorsal aorta; Scale bars, 100 μm.
**b,** Whole-mount immunostaining of E10.0 *Dll4-tdTomato* embryos with the antibodies to RFP (red), major artery endothelial maker CD44 (blue) and pan-endothelial marker CD31 (green). Arrowheads indicate the presumed EP7 located at the branches of internal carotid arteries and in the limb bud capillaries. Arrows indicate the presumed EP6 without Dll4-tdTom expression. Red boxes, reconstructions with a total of 40 μm thickness; yellow boxes, reconstructions with a total of 30 μm thickness. Scale bars, 100 μm.
**c,** Representative FACS plots for cell sorting. Single cell suspensions were prepared from *Dll4-tdTomaoto* and *Unc5b-tdTomato* embryos respectively at different developmental stages as indicated. Cell populations representing different presumed VEC clusters are denoted as colored boxes and were isolated for scRNA-seq.
**d,** RNAscope assay of the indicated mRNAs (red) coupled with immunostaining of Erg (green) on cross sections of E8.5 (upper) and E10.0 (lower) mouse embryos. Note the expression of Cyp26a1 specifically in the aortic VECs at E8.5 but not in the VECs at E10.0. Arrowheads denote $Ptp4a3^{+}$ VECs, which location is restricted to some small vessels, including intersegmental branches from the dorsal aorta, in accordance with that of EP7, but not expressed at major vessels including DAs and ACVs. Images in white boxes show inserts at high magnification. The diagrams to the left indicate the positions of sections. nt, neural tube; DA, dorsal aorta; ACV, anterior cardinal vein. ISA, intersegmental artery. Scale bars, 100 μm.
