# Supplementary material for: Heterogeneity in endothelial cells and widespread venous arterialization during early vascular development in mammals
Source: Cell Res. 2022 Jan 25;32(4):333–48. doi: 10.1038/s41422-022-00615-z (PMC8975889; doi:10.1038/s41422-022-00615-z)
Supplement: Supplementary file 6 — Supplementary information, Fig. S6 [file 41422_2022_615_MOESM6_ESM.pdf]

Fig. S6

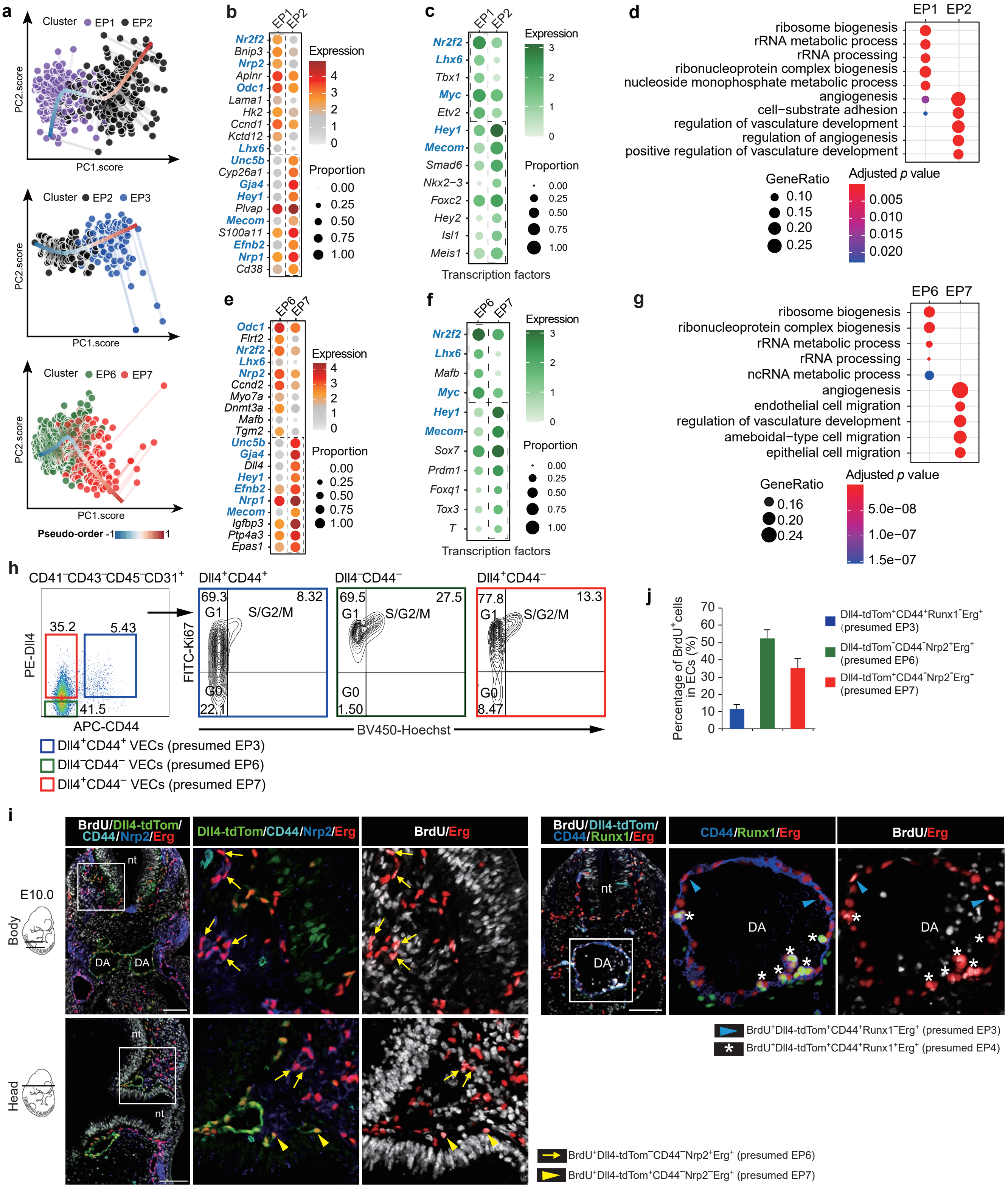

a
Cluster EP1 EP2
Cluster EP2 EP3
Cluster EP6 EP7
PC2.score
PC1.score
Pseudo-order -1 1
b
EP1 EP2
Nr2f2 Bnip3 Nrp2 Aplnr Odc1 Lama1 Hk2 Ccnd1 Kctd12 Lhx6 Unc5b Cyp26a1 Gja4 Hey1 Plvap Mecom S100a11 Efnb2 Nrp1 Cd38
Expression
Proportion
0.00 0.25 0.50 0.75 1.00
c
Nr2f2 Lhx6 Tbx1 Myc Etv2 Hey1 Mecom Smad6 Nkx2−3 Foxc2 Hey2 Isl1 Meis1
Transcription factors
d
EP1 EP2
ribosome biogenesis
rRNA metabolic process
rRNA processing
ribonucleoprotein complex biogenesis
nucleoside monophosphate metabolic process
angiogenesis
cell−substrate adhesion
regulation of vasculature development
regulation of angiogenesis
positive regulation of vasculature development
GeneRatio 0.10 0.15 0.20 0.25
Adjusted p value 0.005 0.010 0.015 0.020
e
EP6 EP7
Odc1 Flrt2 Nr2f2 Lhx6 Nrp2 Ccnd2 Myo7a Dnmt3a Mafb Tgm2 Unc5b Gja4 Dll4 Hey1 Efnb2 Nrp1 Mecom Igfbp3 Ptp4a3 Epas1
f
Nr2f2 Lhx6 Mafb Myc Hey1 Mecom Sox7 Prdm1 Foxq1 Tox3 T
Transcription factors
g
EP6 EP7
ribosome biogenesis
ribonucleoprotein complex biogenesis
rRNA metabolic process
rRNA processing
ncRNA metabolic process
angiogenesis
endothelial cell migration
regulation of vasculature development
ameboidal−type cell migration
epithelial cell migration
GeneRatio 0.16 0.20 0.24
Adjusted p value 5.0e−08 1.0e−07 1.5e−07
h
CD41⁻CD43⁻CD45⁻CD31⁺
PE-Dll4
APC-CD44
35.2 5.43 41.5
Dll4⁺CD44⁺ 69.3 8.32 G1 S/G2/M G0 22.1
Dll4⁻CD44⁻ 69.5 27.5 G1 S/G2/M G0 1.50
Dll4⁺CD44⁻ 77.8 13.3 G1 S/G2/M G0 8.47
FITC-Ki67
BV450-Hoechst
Dll4⁺CD44⁺ VECs (presumed EP3)
Dll4⁻CD44⁻ VECs (presumed EP6)
Dll4⁺CD44⁻ VECs (presumed EP7)
j
Percentage of BrdU⁺cells in ECs (%)
0 10 20 30 40 50 60 70
Dll4-tdTom⁺CD44⁺Runx1⁻Erg⁺ (presumed EP3)
Dll4-tdTom⁻CD44⁻Nrp2⁺Erg⁺ (presumed EP6)
Dll4-tdTom⁺CD44⁻Nrp2⁻Erg⁺ (presumed EP7)
i
BrdU/Dll4-tdTom/CD44/Nrp2/Erg
Dll4-tdTom/CD44/Nrp2/Erg
BrdU/Erg
BrdU/Dll4-tdTom/CD44/Runx1/Erg
CD44/Runx1/Erg
E10.0
Body
Head
nt
DA
BrdU⁺Dll4-tdTom⁺CD44⁺Runx1⁻Erg⁺ (presumed EP3)
BrdU⁺Dll4-tdTom⁺CD44⁺Runx1⁺Erg⁺ (presumed EP4)
BrdU⁺Dll4-tdTom⁻CD44⁻Nrp2⁺Erg⁺ (presumed EP6)
BrdU⁺Dll4-tdTom⁺CD44⁻Nrp2⁻Erg⁺ (presumed EP7)

Fig. S6. Molecular features of different arterial VEC populations.

a, PCA plots with the pseudo-order inferred by fitting a principal curve to the top two dimensions from PCA of EP1 and EP2 (upper), EP2 and EP3 (middle), and EP6 and EP7 (lower).
b, Dot plot showing the average expression levels and cell expressing proportions of top ten DEGs between EP1 and EP2. Dot color indicates the average expression level within a cluster and dot size represents the number of genes within a cluster. The DEGs shared with (e) are indicated in blue.
c, Dot plot showing the differentially expressed transcription factors between EP1 and EP2. The transcription factors shared with (f) are indicated in blue.
d, Dot plot showing the top five enriched Gene Ontology biological process terms for EP1 and EP2. Dot color indicates the statistical significance of the enrichment and dot size represents the fraction of genes annotated to each term.
e, Dot plot showing the differentially expressed top ten genes between EP6 and EP7 which are similar to those between EP2 and EP1 (b). The DEGs shared with (b) are indicated in blue.
f, Dot plot showing the differentially expressed transcription factors between EP6 and EP7 which are similar to those between EP2 and EP1 (c). The transcription factors shared with (c) are indicated in blue.
g, Dot plot showing the enriched Gene Ontology biological process terms of EP6 and EP7 which are similar to those between EP2 and EP1 (d). Dot color indicates statistical significance of the enrichment and dot size represents the fraction of genes annotated to each term.
h, Representative FACS analysis showing the proliferation status of distinct VEC populations. Data are representative of 2 independent experiments. The presumed EP3 (Dll4^{+}CD44^{+}, blue boxes), EP6 (Dll4^{-}CD44^{-}, green boxes), and EP7 (Dll4^{+}CD44^{-}, red boxes) are indicated. Representative plots to the right showing the expression of Hoechst and Ki67 in the indicated VEC populations.
i, Representative immunostaining on cross sections of E10.0 *Dll4-tdTomato* embryos with BrdU injection 0.5 hours prior to cell sample collection. Arrows indicate the BrdU^{+} vein & venous plexus VECs (Dll4-tdTom^{-}CD44^{-}Nrp2^{+}Erg^{+}), yellow arrowheads indicate the BrdU^{+} artery plexus VECs (Dll4-tdTom^{+}CD44^{-}Nrp2^{-}Erg^{+}), blue arrowheads indicate the BrdU^{+} major artery VECs (Dll4-tdTom^{+}CD44^{+}Nrp2^{-}Erg^{+}), and asterisks indicate the BrdU^{+} hemogenic endothelial cells (Dll4-tdTom^{+}CD44^{+}Runx1^{+}Erg^{+}). Images in white boxes show inserts at high magnification. The diagram to the left indicates the positions of sections. nt, neural tube; DA, dorsal aortae. Scale bars, 100 μm.
j, Bar charts showing the proportion of BrdU^{+} VECs in the indicated embryo proper VEC clusters. Notice that the different proliferation status of distinct VEC populations are consistent with the computational (Fig. 3c) and flow cytometric analysis (h). 651 cells of EP3 from 12 sections, 2721 cells of EP6 from 12 sections, and 1778 cells of EP7 from 12 sections were measured respectively.
