# Supplementary material for: Heterogeneity in endothelial cells and widespread venous arterialization during early vascular development in mammals
Source: Cell Res. 2022 Jan 25;32(4):333–48. doi: 10.1038/s41422-022-00615-z (PMC8975889; doi:10.1038/s41422-022-00615-z)
Supplement: Supplementary file 7 — Supplementary information, Fig. S7 [file 41422_2022_615_MOESM7_ESM.pdf]

Fig. S7

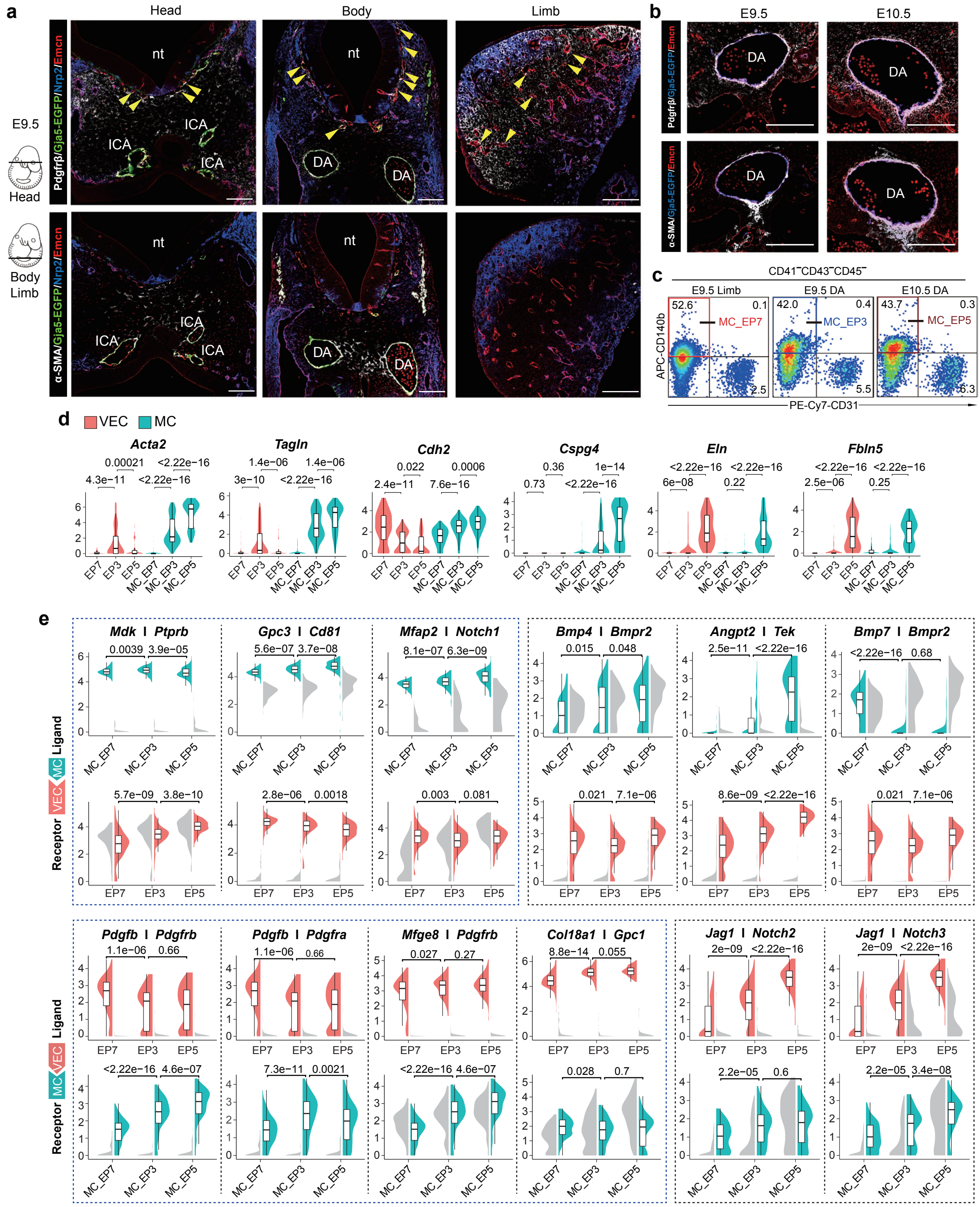

a
Head
Body
Limb
E9.5
Pdgfrβ/Gja5-EGFP/Nrp2/Emcn
α-SMA/Gja5-EGFP/Nrp2/Emcn
nt
ICA
DA
b
E9.5
E10.5
c
CD41⁻CD43⁻CD45⁻
E9.5 Limb
E9.5 DA
E10.5 DA
MC_EP7
MC_EP3
MC_EP5
APC-CD140b
PE-Cy7-CD31
d
VEC
MC
Acta2
Tagln
Cdh2
Cspg4
Eln
Fbln5
e
Mdk | Ptprb
Gpc3 | Cd81
Mfap2 | Notch1
Bmp4 | Bmpr2
Angpt2 | Tek
Bmp7 | Bmpr2
Pdgfb | Pdgfrb
Pdgfb | Pdgfra
Mfge8 | Pdgfrb
Col18a1 | Gpc1
Jag1 | Notch2
Jag1 | Notch3
Ligand
Receptor

Fig. S7. Putative heterologous cellular interactions relevant to mural cell recruitment.

a, b, Immunostaining on cross sections of mouse embryos at different regions and developmental stages as indicated. α-SMAs$^+$ mural cell coverage is clearly detected around DAs and ICAs. In addition to the similar pattern, Pdgfrβ$^+$ mural cell coverage is also detected at the perineural vascular plexus and limb bud capillaries (**a**). Note the increased mural cell recruitment in the DAs of E10.5 compared to E9.5 (**b**). Arrowheads, artery plexus VECs (Nrp2$^-$Emcn$^+$) with Pdgfrβ$^+$ mural cell investment; nt, neural tube. DA, dorsal aorta; ICA, internal carotid artery. Scale bars, 100 μm.

c, Representative FACS plots for CD41$^-$CD43$^-$CD45$^-$CD31$^-$CD144$^-$Pdgfrβ$^+$ mural cells sorting. Single cell suspensions were prepared from mouse embryos at indicated stages and locations. Colored boxes indicate sampling cells for scRNA-seq. DA, dorsal aorta. MC, mural cells.

d, Violin plots with boxplot inside showing the expression levels of the indicated genes in different mural cell populations and the corresponding embryo proper VEC clusters. Wilcoxon Rank Sum test is employed to test the significance of gene expression difference and *P* values are indicated for the comparison. $P < 0.05$ is considered to be statistically significant. MC, mural cells.

e, Paired half-violin plot was designed for illustrating the expression levels of paired ligand (left half) and receptor (right half) genes in the same cell population. These paired half-violin plots showing expression level of the indicated gene pairs in different mural cell populations and VEC populations, which are screened out as heterologous ligand-receptor pairs between different arterial VEC populations and their corresponding mural cell populations referred in (Fig. 3e). Half violins of the putative ligand or receptor providing cell populations were colored by corresponding VEC or MC populations, otherwise colored with grey. The shared (blue dotted line frame) and distinct (black dotted line frame) heterologous ligand-receptor pairs among three arterial vasculatures are indicated. Wilcoxon Rank Sum test is employed to test the significance of gene expression difference and *P* values are indicated for the comparison. $P < 0.05$ is considered to be statistically significant. MC, mural cells.
