# Supplementary material for: Heterogeneity in endothelial cells and widespread venous arterialization during early vascular development in mammals
Source: Cell Res. 2022 Jan 25;32(4):333–48. doi: 10.1038/s41422-022-00615-z (PMC8975889; doi:10.1038/s41422-022-00615-z)
Supplement: Supplementary file 8 — Supplementary information, Fig. S8 [file 41422_2022_615_MOESM8_ESM.pdf]

Fig. S8

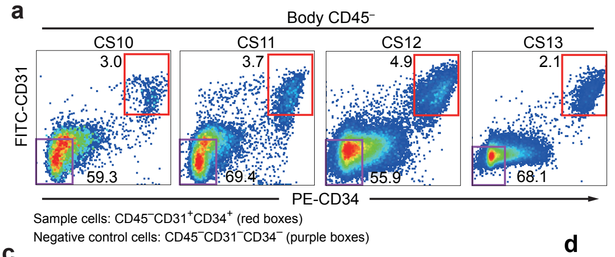

b

ID	Carnegie Stage	Corresponding Stage in Mouse	Cell Number				Total (after quality control)
			Body	Head	Limb	Neg in body	
No. 1	CS10 (22 dpc)	E8.5	58	5	/	24	87 (83)
No. 2	CS11 (24 dpc)	E9.0	96	2	/	26	124 (114)
No. 3	CS12 (26 dpc)	E9.5	96	72	8	32	208 (192)
No. 4	CS12 (26 dpc)	E9.5	168	96	/	24	288 (207)
No. 5	CS13 (28 dpc)	E10.0	160	56	11	32	259 (239)
Total			578	231	19	138	966 (835)

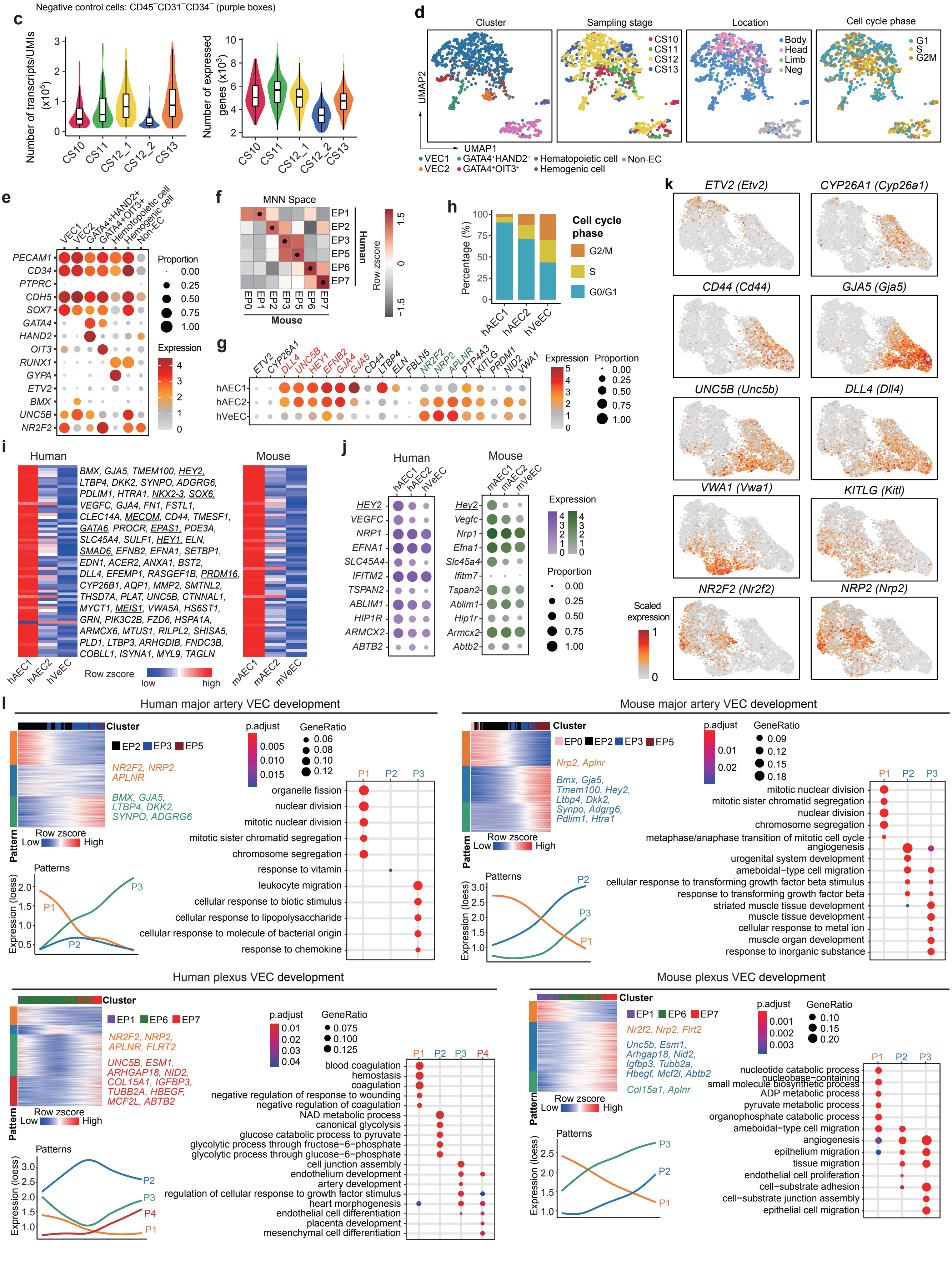

Fig. S8. Single-cell transcriptomic features of VECs from human embryos.

a, Representative FACS plots for cell sorting. Cell populations isolated for scRNA-seq are denoted as colored boxes (red for $CD45^{-}CD31^{+}CD34^{+}$ sample cells and purple for $CD45^{-}CD31^{-}$ $CD34^{-}$ negative control cells derived from the body).
b, Independent experiments and cell number information for scRNA-seq. Neg in body, negative control (non-endothelial) cells derived from the body part.
c, Violin plots showing the number of transcripts (left) and expressed genes (right) in each single cell of different batches.
d, UMAP plots of cells in human VEC dataset, with clusters, sampling stages, locations and cell cycle phases mapped onto it.
e, Dot plot showing the expression of representative marker genes. The size of the dot indicates the percentage of expressing cells within a cluster, and the color indicates the average expression level of cells within a cluster.
f, Correlation analysis in MNN Space validating the assignment accuracy of human VECs in the integration analysis combining human VECs with mouse vascular VECs.
g, Dot plot showing the average and percentage expression of key feature genes distinguishing mouse embryo proper VEC cluster as shown in Fig. 1e in different human VEC populations. The size of the dot indicates the percentage of expressing cells within a cluster, and the color indicates the average expression level of cells within a cluster. Known arterial and venous genes are indicated in red and green, respectively.
h, Constitutions of cells with different cell cycle phases in the indicated human VEC clusters.
i, Heatmaps showing the scaled average expression of conserved genes for hAEC1 and mAEC1. Genes are listed in an order determined by Fisher's combined *P* value from independent *P* values of human and mouse datasets. Transcription factor genes are underlined. For hAEC1/mAEC1, gene names of mouse dataset are omitted, on account of the same spelling with human dataset except for case.
j, Dot plots showing the average and percentage expression of conserved arterial genes satisfying three of four comparisons (two arterial clusters respectively compared to the corresponding venous cluster in both human and mouse). Genes are listed in ascending order of Fisher's combined P value from independent P values of human and mouse datasets. Transcription factor genes are underlined.
k, UMAP plots showing the expression of indicated marker genes on the integrated map.
l, Heatmaps showing the expression changes of pattern genes along development paths as shown in Fig. 5f. Diagrams of pattern changes are shown below. The top ten (if have) conserved genes for each of the three types of arteriovenous VECs included in the pattern genes are indicated using the same color as their expression patterns, with those of major artery VECs and venous VECs shown to the upper and those of arterial plexus VECs and venous VECs shown to the lower. Enriched GO terms for each pattern are shown below.
