# Supplementary material for: Heterogeneity in endothelial cells and widespread venous arterialization during early vascular development in mammals
Source: Cell Res. 2022 Jan 25;32(4):333–48. doi: 10.1038/s41422-022-00615-z (PMC8975889; doi:10.1038/s41422-022-00615-z)
Supplement: Supplementary file 9 — Supplementary information, Fig. S9 [file 41422_2022_615_MOESM9_ESM.pdf]

Fig. S9

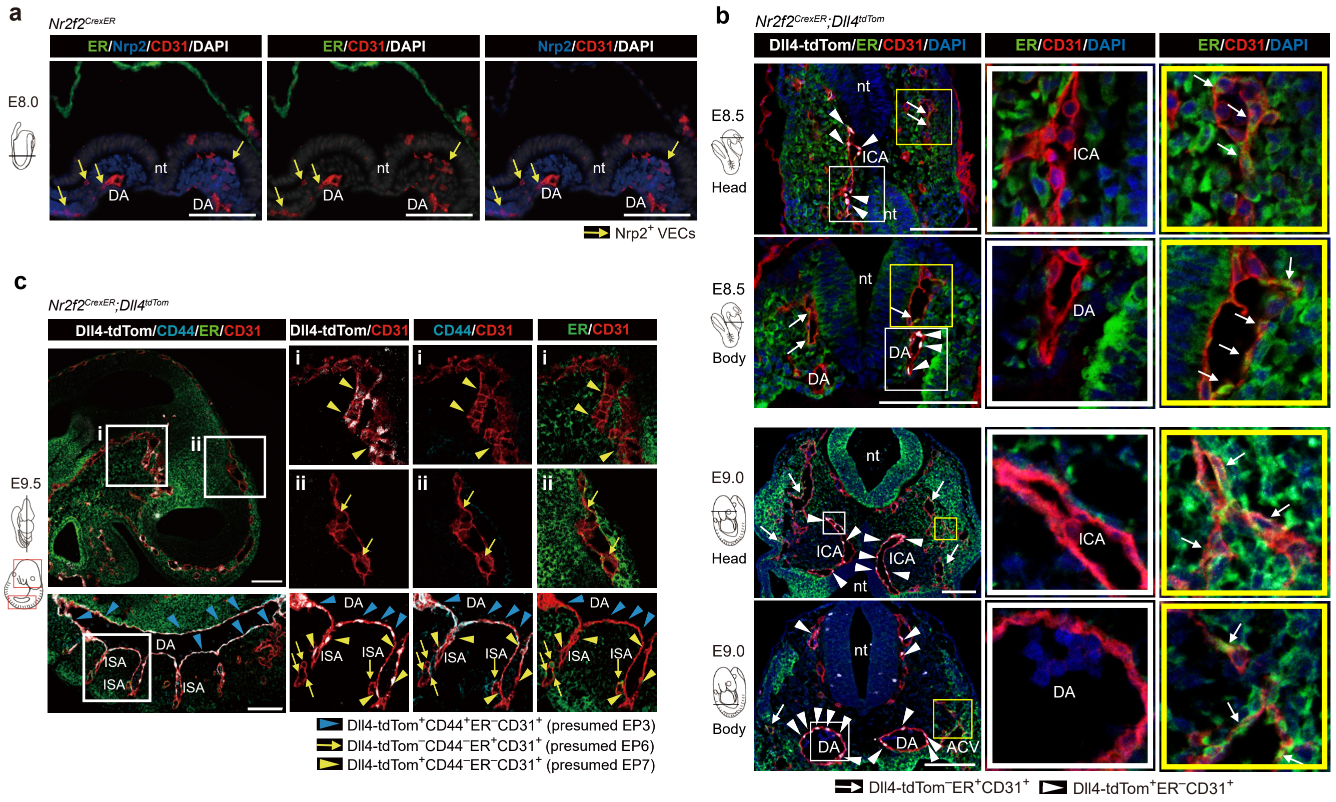

a
Nr2f2CrexER
ER/Nrp2/CD31/DAPI
ER/CD31/DAPI
Nrp2/CD31/DAPI
E8.0
nt
DA
Nrp2+ VECs
b
Nr2f2CrexER;Dll4tdTom
Dll4-tdTom/ER/CD31/DAPI
ER/CD31/DAPI
ER/CD31/DAPI
E8.5
Head
E8.5
Body
E9.0
Head
E9.0
Body
nt
ICA
DA
ACV
Dll4-tdTom−ER+CD31+
Dll4-tdTom+ER−CD31+
c
Nr2f2CrexER;Dll4tdTom
Dll4-tdTom/CD44/ER/CD31
Dll4-tdTom/CD31
CD44/CD31
ER/CD31
E9.5
i
ii
DA
ISA
Dll4-tdTom+CD44+ER−CD31+ (presumed EP3)
Dll4-tdTom−CD44−ER+CD31+ (presumed EP6)
Dll4-tdTom+CD44−ER−CD31+ (presumed EP7)

Fig. S9. Expression of Nr2f2-CrexER in mouse embryos.

a-c, Representative immunostaining on cross sections (E8.0-E9.0) and sagittal sections (E9.5) of *Nr2f2-CrexER* and *Nr2f2-CrexER*;*Dll4-tdTomato* embryos as indicated. At E8.0 (a), expression of ER was not detected in the VECs, in contrast to that of Nrp2 (arrows). From E8.5 to E9.5 (b-c), endothelial expression of ER (arrows) is complementary to that of Dll4-tdTom (arrowheads), in line with the presumed endogenous vascular Nr2f2 expression. Images to the right show inserts at high magnification. The diagrams to the left indicate the positions of sections and imaging. nt, neural tube; DA, dorsal aorta; ICA, internal carotid artery; ISA, intersegmental artery; ACV, anterior cardinal vein. Scale bars, 100 μm.
