# Supplementary material for: Heterogeneity in endothelial cells and widespread venous arterialization during early vascular development in mammals
Source: Cell Res. 2022 Jan 25;32(4):333–48. doi: 10.1038/s41422-022-00615-z (PMC8975889; doi:10.1038/s41422-022-00615-z)
Supplement: Supplementary file 10 — Supplementary information, Fig. S10 [file 41422_2022_615_MOESM10_ESM.pdf]

Fig. S10

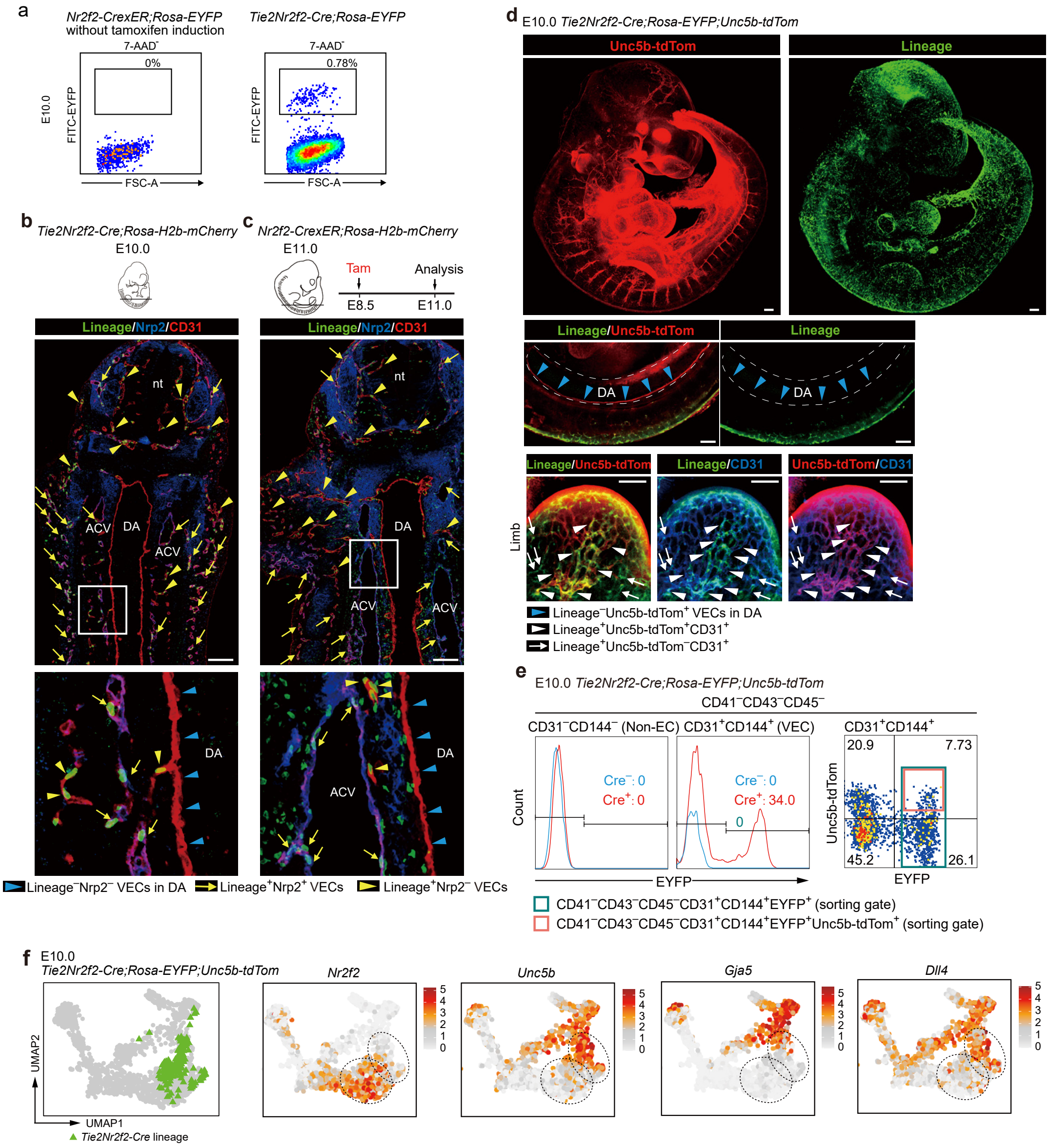
a
Nr2f2-CrexER;Rosa-EYFP without tamoxifen induction
Tie2Nr2f2-Cre;Rosa-EYFP
7-AAD⁻
0%
0.78%
E10.0
FITC-EYFP
FSC-A
b Tie2Nr2f2-Cre;Rosa-H2b-mCherry
E10.0
c Nr2f2-CrexER;Rosa-H2b-mCherry
E11.0
Tam
Analysis
E8.5
E11.0
Lineage/Nrp2/CD31
nt
ACV
DA
Lineage⁻Nrp2⁻ VECs in DA
Lineage⁺Nrp2⁺ VECs
Lineage⁺Nrp2⁻ VECs
d E10.0 Tie2Nr2f2-Cre;Rosa-EYFP;Unc5b-tdTom
Unc5b-tdTom
Lineage
Lineage/Unc5b-tdTom
Lineage/CD31
Unc5b-tdTom/CD31
Limb
Lineage⁻Unc5b-tdTom⁺ VECs in DA
Lineage⁺Unc5b-tdTom⁺CD31⁺
Lineage⁺Unc5b-tdTom⁻CD31⁺
e E10.0 Tie2Nr2f2-Cre;Rosa-EYFP;Unc5b-tdTom
CD41⁻CD43⁻CD45⁻
CD31⁻CD144⁻ (Non-EC)
CD31⁺CD144⁺ (VEC)
CD31⁺CD144⁺
Count
Cre⁻: 0
Cre⁺: 0
Cre⁺: 34.0
EYFP
Unc5b-tdTom
20.9
7.73
45.2
26.1
CD41⁻CD43⁻CD45⁻CD31⁺CD144⁺EYFP⁺ (sorting gate)
CD41⁻CD43⁻CD45⁻CD31⁺CD144⁺EYFP⁺Unc5b-tdTom⁺ (sorting gate)
f E10.0
Tie2Nr2f2-Cre;Rosa-EYFP;Unc5b-tdTom
UMAP1
UMAP2
Tie2Nr2f2-Cre lineage
Nr2f2
Unc5b
Gja5
Dll4
5
4
3
2
1
0

Fig. S10. The arterial fate of Nr2f2-expressing VECs revealed by genetic lineage tracing.

a, Representative FACS plots showing the undetectable EYFP expression in E10.0 *Nr2f2-CrexER;Rosa-EYFP* embryos without tamoxifen induction, with the littermate *Tie2Nr2f2-Cre;Rosa-EYFP* embryos as the positive control.

b, Representative immunostaining on cross sections of E10.0 *Tie2Nr2f2-Cre;Rosa-H2b-mCherry* embryos. The lineage-labeled cells are confined to VECs and widely distributed throughout the vasculature including $Nrp2^{+}$ venous VECs (yellow arrows) and the presumed artery plexus VECs ($Nrp2^{-}$, yellow arrowheads), but seldom in the aortic VECs (blue arrowheads). Images at the bottom show inserts at high magnification. The diagram above indicates the position of the sections. nt, neural tube; DA, dorsal aorta; ACV, anterior cardinal vein. Scale bars, 100 μm.

c, Representative immunostaining on cross sections of E11.0 *Nr2f2-CrexER;Rosa-H2b-mCherry* embryos after single dose of tamoxifen induction at E8.5. Note the lineage-labeled cells widely distributed throughout the vasculature including $Nrp2^{+}$ venous VECs (yellow arrows) and the presumed artery plexus VECs ($Nrp2^{-}$, yellow arrowheads), but seldom in the aortic VECs (blue arrowheads). Images at the bottom show inserts at high magnification. The diagrams to the upper indicate the position of the sections and the inducible tracing strategy. nt, neural tube; DA, dorsal aorta; ACV, anterior cardinal vein. Scale bars, 100 μm.

d, Whole-mount immunostaining of E10.0 *Tie2Nr2f2-Cre;Rosa-EYFP;Unc5b-tdTomato* embryos with antibodies to RFP (red, Unc5b-tdTom), GFP (green, *Tie2Nr2f2-Cre* lineage) and pan-endothelial marker CD31 (blue). Note the contribution of *Tie2Nr2f2-Cre* lineage to the limb bud plexus VECs including the Unc5b-$tdTom^{+}$ arterial VECs (white arrowheads) and the presumed venous VECs (Unc5b-$tdTom^{-}$, white arrows), but not to the aorta (blue arrowheads). Representative monolayer images are shown for the aorta and limb bud regions, respectively. Dashed lines indicate the boundary of the aorta. DA, dorsal aorta. Scale bars, 100 μm.

e, Representative FACS plots for analysis and cell sorting. Single cell suspensions were prepared from E10.0 *Tie2Nr2f2-Cre;Rosa-EGFP;Unc5b-tdTomato* embryos. Histograms show that the lineage labeling is confined to the immunophenotypic VECs. Cell populations isolated for scRNA-seq are denoted as colored boxes (red for $lineage^{+}$Unc5b-$tdTom^{+}$ VECs and blue for $lineage^{+}$ VECs). Littermate *Rosa-EYFP* embryos are used as the negative control.

f, UMAP plots of the integration data of sequenced *Tie2Nr2f2-Cre* lineage labeled cells and original VECs with the expression of indicated arteriovenous marker genes mapped on. Note that most lineage and Unc5b double-positive VECs show clear arterial genes but a loss of Nr2f2 expression. Dashed outlines of EP6 and EP7 are indicated.
