# Supplementary material for: Heterogeneity in endothelial cells and widespread venous arterialization during early vascular development in mammals
Source: Cell Res. 2022 Jan 25;32(4):333–48. doi: 10.1038/s41422-022-00615-z (PMC8975889; doi:10.1038/s41422-022-00615-z)
Supplement: Supplementary file 11 — Supplementary information, Fig. S11 [file 41422_2022_615_MOESM11_ESM.pdf]

Fig. S11

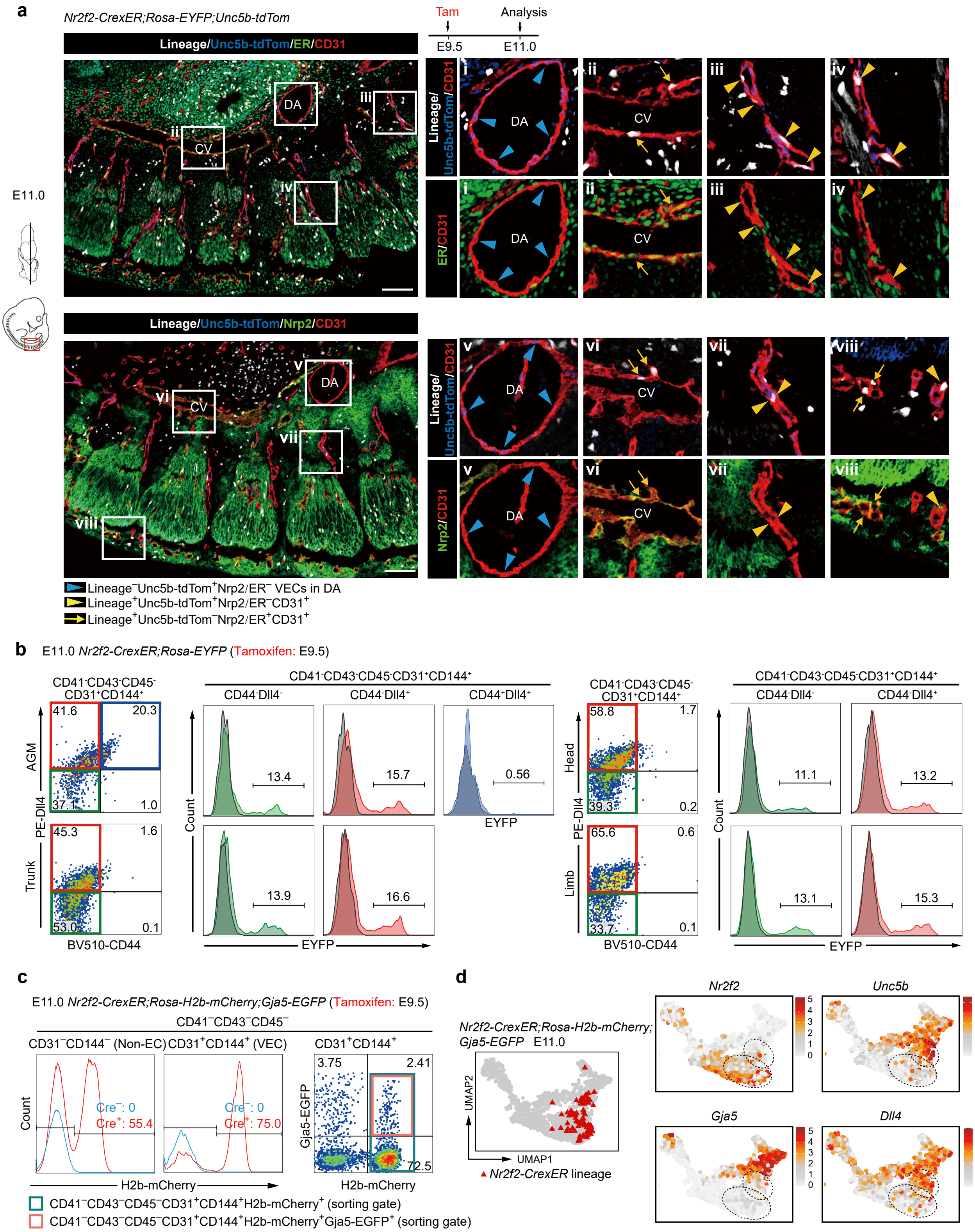
a
Nr2f2-CrexER;Rosa-EYFP;Unc5b-tdTom
Tam
Analysis
E9.5
E11.0
Lineage/Unc5b-tdTom/ER/CD31
E11.0
DA
CV
i
ii
iii
iv
Lineage/Unc5b-tdTom/CD31
ER/CD31
Lineage/Unc5b-tdTom/Nrp2/CD31
v
vi
vii
viii
Nrp2/CD31
Lineage⁻Unc5b-tdTom⁺Nrp2/ER⁻ VECs in DA
Lineage⁺Unc5b-tdTom⁺Nrp2/ER⁻CD31⁺
Lineage⁺Unc5b-tdTom⁻Nrp2/ER⁺CD31⁺
b
E11.0 Nr2f2-CrexER;Rosa-EYFP (Tamoxifen: E9.5)
CD41⁻CD43⁻CD45⁻ CD31⁺CD144⁺
AGM
Trunk
PE-Dll4
BV510-CD44
41.6
20.3
37.1
1.0
45.3
1.6
53.0
0.1
CD41⁻CD43⁻CD45⁻CD31⁺CD144⁺
CD44⁻Dll4⁻
CD44⁻Dll4⁺
CD44⁺Dll4⁺
Count
13.4
15.7
0.56
13.9
16.6
EYFP
Head
Limb
58.8
1.7
39.3
0.2
65.6
0.6
33.7
0.1
11.1
13.2
13.1
15.3
c
E11.0 Nr2f2-CrexER;Rosa-H2b-mCherry;Gja5-EGFP (Tamoxifen: E9.5)
CD41⁻CD43⁻CD45⁻
CD31⁻CD144⁻ (Non-EC)
CD31⁺CD144⁺ (VEC)
CD31⁺CD144⁺
Count
Cre⁻: 0
Cre⁺: 55.4
Cre⁺: 75.0
H2b-mCherry
Gja5-EGFP
3.75
2.41
72.5
CD41⁻CD43⁻CD45⁻CD31⁺CD144⁺H2b-mCherry⁺ (sorting gate)
CD41⁻CD43⁻CD45⁻CD31⁺CD144⁺H2b-mCherry⁺Gja5-EGFP⁺ (sorting gate)
d
Nr2f2-CrexER;Rosa-H2b-mCherry;
Gja5-EGFP E11.0
UMAP1
UMAP2
Nr2f2-CrexER lineage
Nr2f2
Unc5b
Gja5
Dll4

Fig. S11. Capillary arterialization from venous-featured plexus VECs in mid-gestational embryos revealed by genetic lineage tracing.

a, Representative immunostaining on sagittal sections of E11.0 *Nr2f2-CrexER;Rosa-EYFP;Unc5b-tdTomato* embryos after single dose of tamoxifen induction at E9.5. The *Nr2f2-CrexER* lineage-labeled VECs are observed in the Unc5b-tdTom^{+}Nrp2/ER^{-} artery plexus VECs (yellow arrowheads) in addition to Unc5b-tdTom^{-}Nrp2/ER^{+} venous VECs (yellow arrows), but not in the DA (blue arrowheads). Images to the right show inserts at high magnification. The diagrams to the left indicate the positions of the sections and imaging. DA, dorsal aorta. CV, cardinal vein. Scale bars, 100 μm.

b, Representative FACS analysis of different regions of E11.0 *Nr2f2-CreER;Rosa-EYFP* embryos after single dose of tamoxifen induction at E9.5. Data are representative of 2 independent experiments. The presumed EP6 (vein & venous plexus VECs, green boxes), EP7 (arterial plexus VECs, red boxes), and major artery VECs (CD44^{+}Dll4^{+} in AGM region, blue box) are indicated. Histograms to the right indicate the EYFP labelling in different populations. Littermate Rosa-EYFP embryos are used as the negative controls. AGM, aorta-gonad-mesonephros.

c, Representative FACS plots for analysis and cell sorting. Single cell suspensions were prepared from E11.0 *Nr2f2-CrexER;Rosa-H2b-mCherry;Gja5-EGFP* embryos after single dose of tamoxifen induction at E9.5. Histograms show the lineage labeling in both non-ECs and VECs. Cell populations isolated for scRNA-seq are donated as colored boxes (red for lineage^{+}Gja5-EGFP^{+} VECs and blue for lineage^{+} VECs). Littermate *Rosa-H2b-mCherry* embryos are used as the negative control.

d, UMAP plots of the integration data of sequenced *Nr2f2-CrexER* lineage labeled cells and original VECs with the expression of indicated arteriovenous marker genes mapped on. Note that most lineage and Gja5 double-positive VECs show clear arterial genes but a loss of Nr2f2 expression. Dashed outlines of EP6 and EP7 are indicated.
